# Supplementary material for: Development and external validation of prediction algorithms to improve early diagnosis of cancer
Source: Nat Commun. 2025 May 7;16:3660. doi: 10.1038/s41467-025-57990-5 (PMC12059126; doi:10.1038/s41467-025-57990-5)
Supplement: Supplementary file 1 — Supplementary Information [file 41467_2025_57990_MOESM1_ESM.pdf]

# Supplementary Tables

**Development and external validation of prediction algorithms to improve early diagnosis of cancer**

**Authors**
Hippisley-Cox J*, Coupland CA.

**Affiliations**
1 Professor of Clinical Epidemiology & Predictive Medicine. Queen Mary University of London, UK. http://orcid.org/0000-0002-2479-7283
2 Senior Research Fellow, University of Oxford, UK. https://orcid.org/0000-0002-2327-3306

**Corresponding author:** julia.hippisley-cox@qmul.ac.uk

This PDF includes
- Tables 1-16
- Figures 1-26
- Information on sample size calculations

Supplementary Table 1. Numbers of men and women meeting the inclusion and exclusion criteria for the derivation cohort and each validation cohort.

| | ***QResearch derivation*** | ***QResearch validation*** | ***CPRD validation*** | ***QResearch derivation*** | ***QResearch validation*** | ***CPRD validation*** |
|---|---|---|---|---|---|---|
| | **Women (col %)** | **Women (col %)** | **Women (col %)** | **Men(col%)** | **Men (col%)** | **Women (col %)** |
| total number of practices | 1,071 | 357 | 398 | 1,071 | 357 | 398 |
| total population aged 18-84 | 5,984,513 (100) | 2,149,197 (100) | 1,910,399 | 5,752,370 (100) | 2,067,128 (100) | 1,815,127 |
| **Exclusions** | | | | | | |
| no Townsend recorded | 101,465 (1.7) | 35,018 (1.6) | n/a | 79,745 (1.4) | 27,334 (1.3) | n/a |
| at least one red flag symptom in 12 months before cohort entry | 382,174 (6.4) | 135,133 (6.3) | 120,522 (6.3) | 235,354 (4.1) | 81,951 (4.0) | 74,491 (4.10) |
| Less than 2 years follow-up | 1,878,085 (31.4) | 702,031 (32.7) | 416,871 (21.8) | 1,595,553 (27.7) | 597,674 (28.9) | 376,916 (20.8) |
| **Included** | | | | | | |
| Target group after exclusions | 3,622,789 (60.5) | 1,277,015 (59.4) | 1,373,006 (71.9) | 3,841,718 (66.8) | 1,360,169 (65.8) | 1,363,720 (75.1) |
| Total incident cancers during 2 years' follow-up | 64,233 | 22,105 | 16,590 | 65,482 | 22,879 | 15,738 |

**Supplementary Table 2: Baseline characteristics of men and women aged 18-84 years in the English QResearch derivation and validation cohorts. Values are numbers (column%) unless indicated otherwise.**

| | *QResearch* derivation women | *QResearch* validation women | *CPRD* validation women | *QResearch* derivation men | *QResearch* validation men | *CPRD* validation men |
|---|---|---|---|---|---|---|
| total | 3622789 | 1277015 | 1373006 | 3841718 | 1360169 | 1363720 |
| mean age (SD) | 44.9 (17.9) | 44.6 (18.0) | 48.5 (18.0) | 44.3 (17.1) | 44.1 (17.2) | 47.5 (17.4) |
| mean Townsend (SD) | 0.5 (3.1) | 0.5 (3.2) | 0.0 (0.0) | 0.6 (3.1) | 0.6 (3.2) | 0.0 (0.0) |
| mean BMI (SD) | 26.9 (6.2) | 26.8 (6.2) | 133.1 (12.1) | 27.3 (5.2) | 27.3 (5.2) | 147.6 (14.2) |
| mean HB (SD) | 131.5 (12.4) | 131.5 (12.4) | 8.9 (5.0) | 146.9 (14.1) | 146.8 (14.1) | 11.4 (6.5) |
| mean bilirubin (SD) | 8.9 (5.1) | 9.0 (5.1) | 275.0 (70.4) | 11.4 (6.4) | 11.4 (6.4) | 244.8 (66.8) |
| mean platelet (SD) | 269.5 (69.8) | 268.2 (69.6) | 42.0 (4.3) | 241.1 (65.7) | 240.2 (65.7) | 42.8 (4.4) |
| mean neutrophil (SD) | 4.2 (1.9) | 4.2 (1.9) | 4.3 (1.9) | 4.2 (1.9) | 4.2 (1.9) | 4.3 (1.8) |
| mean lymphocyte (SD) | 2.1 (0.9) | 2.1 (0.9) | 2.1 (0.8) | 2.1 (1.1) | 2.1 (1.1) | 2.1 (1.0) |
| mean albumin (SD) | 42.2 (4.1) | 42.3 (4.1) | 79.3 (33.0) | 43.0 (4.2) | 43.1 (4.2) | 79.3 (33.4) |
| mean alkaline phosphophatase (SD) | 79.0 (35.1) | 78.6 (34.0) | 27.5 (5.6) | 80.2 (35.8) | 79.8 (35.0) | 28.1 (4.9) |
| | | | | | | |
| **Ethnicity** | | | | | | |
| White | 1909660(52.7) | 670729 (52.5) | 431376 (31.4) | 1870458(48.7) | 655747 (48.2) | 414206 (30.4) |
| Indian | 112558 (3.1) | 35353 (2.8) | 3725 (0.3) | 129706 (3.4) | 42315 (3.1) | 5136 (0.4) |
| Pakistani | 70054 (1.9) | 18703 (1.5) | 3208 (0.2) | 84342 (2.2) | 22961 (1.7) | 4077 (0.3) |
| Bangladeshi | 38716 (1.1) | 15124 (1.2) | 967 (0.1) | 47999 (1.2) | 18688 (1.4) | 1399 (0.1) |
| Other Asian | 82110 (2.3) | 26737 (2.1) | 3165 (0.2) | 84431 (2.2) | 25905 (1.9) | 2675 (0.2) |
| Caribbean | 40903 (1.1) | 15444 (1.2) | 377 (0.0) | 37762 (1.0) | 14381 (1.1) | 345 (0.0) |
| Black African | 95415 (2.6) | 39923 (3.1) | 3048 (0.2) | 98852 (2.6) | 41359 (3.0) | 3739 (0.3) |
| Chinese | 53693 (1.5) | 14264 (1.1) | 3448 (0.3) | 43876 (1.1) | 11804 (0.9) | 3097 (0.2) |
| Other | 162493 (4.5) | 59412 (4.7) | 7397 (0.5) | 162376 (4.2) | 60764 (4.5) | 7674 (0.6) |
| not recorded | 1057187(29.2) | 381326 (29.9) | 916295 (66.7) | 1281916(33.4) | 466245 (34.3) | 921372 (67.6) |
| | | | | | | |
| **Smoking status** | | | | | | |
| nonsmoker | 2190621(60.5) | 784168 (61.4) | 618077 (45.0) | 1881690(49.0) | 674289 (49.6) | 547614 (40.2) |
| ex-smoker | 639178 (17.6) | 220803 (17.3) | 254369 (18.5) | 788647 (20.5) | 275302 (20.2) | 278308 (20.4) |
| light smoker | 418867 (11.6) | 146106 (11.4) | 63426 (4.6) | 598051 (15.6) | 209634 (15.4) | 56158 (4.1) |
| moderate smoker | 95114 (2.6) | 32254 (2.5) | 95306 (6.9) | 137925 (3.6) | 47414 (3.5) | 116788 (8.6) |
| heavy smoker | 32890 (0.9) | 11237 (0.9) | 35058 (2.6) | 73493 (1.9) | 25450 (1.9) | 60379 (4.4) |
| not recorded | 246119 (6.8) | 82447 (6.5) | 306770 (22.3) | 361912 (9.4) | 128080 (9.4) | 304473 (22.3) |
| | | | | | | |
| **Alcohol status** | | | | | | |
| non drinker | 1812244(50.0) | 642391 (50.3) | 227544 (16.6) | 1627240(42.4) | 574725 (42.3) | 154012 (11.3) |
| Trivial <1u/day | 628130 (17.3) | 227435 (17.8) | 393461 (28.7) | 511950 (13.3) | 186273 (13.7) | 258007 (18.9) |
| Light 1-2u/day | 238654 (6.6) | 79429 (6.2) | 227204 (16.5) | 287031 (7.5) | 100298 (7.4) | 279840 (20.5) |
| Moderate 3-6u/day | 112466 (3.1) | 38098 (3.0) | 24979 (1.8) | 283221 (7.4) | 99093 (7.3) | 89435 (6.6) |
| Heavy 7-9u/day | 5354 (0.1) | 1957 (0.2) | 2587 (0.2) | 26550 (0.7) | 9092 (0.7) | 11699 (0.9) |
| Very Heavy >9u/day | 9242 (0.3) | 2526 (0.2) | 4379 (0.3) | 24652 (0.6) | 7483 (0.6) | 15481 (1.1) |
| not recorded | 816699 (22.5) | 285179 (22.3) | 492852 (35.9) | 1081074(28.1) | 383205 (28.2) | 555246 (40.7) |

| | | | | | | |
|---|---|---|---|---|---|---|
| **Values recorded** | | | | | | |
| BMI | 1728424(47.7) | 615409 (48.2) | 420387 (30.6) | 1415516(36.8) | 507034 (37.3) | 308094 (22.6) |
| HB | 1559943(43.1) | 545269 (42.7) | 467740 (34.1) | 1147505(29.9) | 397329 (29.2) | 395410 (29.0) |
| bilirubin | 1378849(38.1) | 478996 (37.5) | 503207 (36.7) | 1180911(30.7) | 408916 (30.1) | 368430 (27.0) |
| platelet | 1563327(43.2) | 545445 (42.7) | 433269 (31.6) | 1151720(30.0) | 398168 (29.3) | 363284 (26.6) |
| neutrophil | 1558858 43.0) | 543198 (42.5) | 490879 (35.8) | 1147694(29.9) | 396617 (29.2) | 358476 (26.3) |
| lymphocyte | 1557563(43.0) | 541656 (42.4) | 487243 (35.5) | 1146078(29.8) | 395205 (29.1) | 355864 (26.1) |
| albumin | 1363228(37.6) | 478613 (37.5) | 472673 (34.4) | 1149327(29.9) | 402155 (29.6) | 397471 (29.1) |
| alkaline phosphatase | 1403482(38.7) | 487839 (38.2) | 507475 (37.0) | 1187780(30.9) | 411274 (30.2) | 416817 (30.6) |
| **Family history (FH)** | | | | | | |
| FH breast cancer | 205540 (5.7) | 72004 (5.6) | 35 (0.0) | 48558 (1.3) | 16109 (1.2) | 22 (0.0) |
| FH bowel cancer | 82357 (2.3) | 28745 (2.3) | 29769 (2.2) | 60183 (1.6) | 20422 (1.5) | 4406 (0.3) |
| FH ovarian cancer | 14239 (0.4) | 4751 (0.4) | 12021 (0.9) | 2287 (0.1) | 747 (0.1) | 8170 (0.6) |
| FH prostate cancer | 11242 (0.3) | 3611 (0.3) | 2624 (0.2) | 28924 (0.8) | 10069 (0.7) | 299 (0.0) |
| FH lung cancer | 33414 (0.9) | 9836 (0.8) | 710 (0.1) | 26579 (0.7) | 7760 (0.6) | 2853 (0.2) |
| FH blood cancer | 997 (0.0) | 388 (0.0) | 3437 (0.3) | 1176 (0.0) | 395 (0.0) | 2469 (0.2) |
| **Comorbidity** | | | | | | |
| chronic pancreatitis | 1810 (0.0) | 619 (0.0) | 720 (0.1) | 3438 (0.1) | 1186 (0.1) | 1350 (0.1) |
| COPD | 61712 (1.7) | 21110 (1.7) | 26881 (2.0) | 70917 (1.8) | 24450 (1.8) | 27148 (2.0) |
| type 2 diabetes | 151045 (4.2) | 52392 (4.1) | 48910 (3.6) | 211853 (5.5) | 73549 (5.4) | 67971 (5.0) |
| endometrial hyperplasia/polyp | 21394 (0.6) | 7593 (0.6) | 5271 (0.4) | n/a | n/a | n/a |
| liver cirrhosis | 5420 (0.1) | 1948 (0.2) | 2256 (0.2) | 6230 (0.2) | 2214 (0.2) | 2325 (0.2) |
| Hepatitis C | 4957 (0.1) | 1615 (0.1) | 1177 (0.1) | 10303 (0.3) | 3233 (0.2) | 2288 (0.2) |
| Hepatitis B | 8822 (0.2) | 3161 (0.2) | 1193 (0.1) | 9456 (0.2) | 3346 (0.2) | 1667 (0.1) |
| HIV/AIDs | 4113 (0.1) | 1607 (0.1) | 424 (0.0) | 9662 (0.3) | 3528 (0.3) | 1310 (0.1) |
| **General symptoms** | | | | | | |
| erectile dysfunction | n/a | n/a | n/a | 51896 (1.4) | 18423 (1.4) | 17725 (1.3) |
| constipation | 41432 (1.1) | 14299 (1.1) | 11603 (0.8) | 26850 (0.7) | 9391 (0.7) | 7340 (0.5) |
| cough | 204061 (5.6) | 71164 (5.6) | 84119 (6.1) | 159332 (4.1) | 55426 (4.1) | 63623 (4.7) |
| urinary frequency | 31505 (0.9) | 10915 (0.9) | 6359 (0.5) | 16014 (0.4) | 5519 (0.4) | 4498 (0.3) |
| urinary retention | 1432 (0.0) | 480 (0.0) | 253 (0.0) | 5651 (0.1) | 1983 (0.1) | 1409 (0.1) |
| nocturia | 3568 (0.1) | 1674 (0.1) | 811 (0.1) | 7780 (0.2) | 2684 (0.2) | 2518 (0.2) |
| itching | 15094 (0.4) | 5268 (0.4) | 5878 (0.4) | 8995 (0.2) | 3219 (0.2) | 3392 (0.2) |
| change in bowels | 10068 (0.3) | 3500 (0.3) | 2556 (0.2) | 8586 (0.2) | 3058 (0.2) | 2042 (0.1) |
| bruising | 6121 (0.2) | 2105 (0.2) | 1667 (0.1) | 2259 (0.1) | 786 (0.1) | 651 (0.0) |
| back pain | 219420 (6.1) | 75651 (5.9) | 63642 (4.6) | 172518 (4.5) | 59356 (4.4) | 50547 (3.7) |
| hoarseness | 8514 (0.2) | 3048 (0.2) | 2274 (0.2) | 4661 (0.1) | 1651 (0.1) | 1139 (0.1) |
| flatulence | 40048 (1.1) | 13802 (1.1) | 8739 (0.6) | 16601 (0.4) | 5580 (0.4) | 3267 (0.2) |
| abdominal mass | 5133 (0.1) | 1633 (0.1) | 779 (0.1) | 4503 (0.1) | 1382 (0.1) | 713 (0.1) |
| dark urine | 2580 (0.1) | 887 (0.1) | 33 (0.0) | 1979 (0.1) | 713 (0.1) | 49 (0.0) |
| **Alarm symptoms** | | | | | | |
| appetite loss | 20711 (0.6) | 6405 (0.5) | 2293 (0.2) | 14990 (0.4) | 4719 (0.3) | 1909 (0.1) |
| weight loss | 32900 (0.9) | 11146 (0.9) | 7156 (0.5) | 34045 (0.9) | 11466 (0.8) | 7345 (0.5) |

| | | | | | | |
|---|---|---|---|---|---|---|
| abdominal distension | 41502 (1.1) | 14373 (1.1) | 9695 (0.7) | 17050 (0.4) | 5836 (0.4) | 3529 (0.3) |
| abdominal pain | 242159 (6.7) | 84176 (6.6) | 63193 (4.6) | 181651 (4.7) | 62791 (4.6) | 51031 (3.7) |
| dysphagia | 20830 (0.6) | 7489 (0.6) | 5199 (0.4) | 20679 (0.5) | 7225 (0.5) | 5244 (0.4) |
| heartburn | 94113 (2.6) | 32380 (2.5) | 30847 (2.2) | 82720 (2.2) | 28730 (2.1) | 26711 (2.0) |
| indigestion | 62435 (1.7) | 20477 (1.6) | 16476 (1.2) | 52857 (1.4) | 17627 (1.3) | 13898 (1.0) |
| haematemesis | 7096 (0.2) | 2497 (0.2) | 2428 (0.2) | 10345 (0.3) | 3622 (0.3) | 3542 (0.3) |
| rectal bleeding | 56450 (1.6) | 19768 (1.5) | 14315 (1.0) | 68190 (1.8) | 23974 (1.8) | 17341 (1.3) |
| haematuria | 41837 (1.2) | 14628 (1.1) | 8404 (0.6) | 49099 (1.3) | 17345 (1.3) | 12060 (0.9) |
| haemoptysis | 8727 (0.2) | 3020 (0.2) | 2466 (0.2) | 13677 (0.4) | 4637 (0.3) | 3749 (0.3) |
| lump in the neck | 23604 (0.7) | 8394 (0.7) | 5352 (0.4) | 16007 (0.4) | 5624 (0.4) | 3598 (0.3) |
| thrombosis | 27075 (0.7) | 9429 (0.7) | 7832 (0.6) | 26804 (0.7) | 9450 (0.7) | 8495 (0.6) |
| night sweats | 13369 (0.4) | 4815 (0.4) | 2801 (0.2) | 7910 (0.2) | 2653 (0.2) | 1651 (0.1) |
| breast lump | 134892 (3.7) | 47018 (3.7) | 31434 (2.3) | n/a | n/a | n/a |
| breast pain | 71647 (2.0) | 25315 (2.0) | 18035 (1.3) | n/a | n/a | n/a |
| breast skin/nipple changes | 19519 (0.5) | 6682 (0.5) | 4235 (0.3) | n/a | n/a | n/a |
| inter-menstrual bleeding | 43002 (1.2) | 14467 (1.1) | 10548 (0.8) | n/a | n/a | n/a |
| postmenopausal bleeding | 30907 (0.9) | 10724 (0.8) | 9725 (0.7) | n/a | n/a | n/a |
| post coital bleeding | 22471 (0.6) | 7892 (0.6) | 5888 (0.4) | n/a | n/a | n/a |
| testicular pain | n/a | n/a | n/a | 34134 (0.9) | 12191 (0.9) | 5671 (0.4) |
| testicular lump | n/a | n/a | n/a | 45667 (1.2) | 16117 (1.2) | 11477 (0.8) |

**Supplementary Table 3. Crude incidence rate of each cancer type per 1000 person years (95% CI) in the QResearch derivation cohort by data source. Percent of data on GP record only is the number of cases identified on the GP record as a proportion of all the cases identified on any of the linked data sources.**

| *outcome* | | *GP record* | *GP or Mortality record* | *GP, hospital or mortality record* | *GP, hospital or mortality or cancer registry record* | *% cases recorded on GP data* |
|---|---|---|---|---|---|---|
| Lung | female | 0.60 (0.58 to 0.62) | 0.66 (0.65 to 0.68) | 0.76 (0.74 to 0.78) | 0.77 (0.75 to 0.78) | 78 |
| | male | 0.71 (0.69 to 0.73) | 0.82 (0.80 to 0.83) | 0.90 (0.88 to 0.92) | 0.91 (0.89 to 0.93) | 78 |
| | total | 0.65 (0.64 to 0.67) | 0.74 (0.73 to 0.75) | 0.83 (0.82 to 0.85) | 0.84 (0.83 to 0.85) | 78 |
| Colorectal | female | 0.54 (0.53 to 0.56) | 0.29 (0.28 to 0.30) | 0.68 (0.67 to 0.70) | 0.70 (0.68 to 0.72) | 77 |
| | male | 0.70 (0.69 to 0.72) | 0.39 (0.38 to 0.41) | 0.86 (0.84 to 0.88) | 0.88 (0.86 to 0.90) | 80 |
| | total | 0.62 (0.61 to 0.63) | 0.34 (0.33 to 0.35) | 0.77 (0.76 to 0.79) | 0.79 (0.78 to 0.80) | 79 |
| Gastro-oesophageal | female | 0.17 (0.16 to 0.18) | 0.17 (0.16 to 0.18) | 0.21 (0.20 to 0.22) | 0.22 (0.21 to 0.23) | 77 |
| | male | 0.42 (0.41 to 0.43) | 0.41 (0.40 to 0.43) | 0.48 (0.47 to 0.50) | 0.49 (0.47 to 0.50) | 86 |
| | total | 0.30 (0.29 to 0.30) | 0.29 (0.28 to 0.30) | 0.35 (0.34 to 0.36) | 0.36 (0.35 to 0.36) | 83 |
| Pancreas | female | 0.16 (0.15 to 0.17) | 0.21 (0.20 to 0.22) | 0.22 (0.21 to 0.23) | 0.22 (0.21 to 0.23) | 72 |
| | male | 0.19 (0.18 to 0.20) | 0.24 (0.23 to 0.26) | 0.26 (0.25 to 0.27) | 0.26 (0.25 to 0.27) | 73 |
| | total | 0.18 (0.17 to 0.18) | 0.23 (0.22 to 0.23) | 0.24 (0.23 to 0.25) | 0.24 (0.23 to 0.25) | 73 |
| Ovarian | female | 0.24 (0.23 to 0.25) | 0.18 (0.17 to 0.19) | 0.32 (0.31 to 0.34) | 0.35 (0.34 to 0.36) | 69 |
| Renal | female | 0.29 (0.27 to 0.30) | 0.15 (0.14 to 0.15) | 0.39 (0.38 to 0.40) | 0.40 (0.39 to 0.41) | 71 |
| | male | 0.76 (0.74 to 0.78) | 0.32 (0.31 to 0.33) | 1.01 (0.99 to 1.03) | 1.03 (1.01 to 1.05) | 74 |
| | total | 0.53 (0.52 to 0.54) | 0.23 (0.23 to 0.24) | 0.70 (0.69 to 0.72) | 0.72 (0.71 to 0.73) | 73 |
| Breast | female | 2.24 (2.21 to 2.27) | 0.49 (0.48 to 0.51) | 2.42 (2.38 to 2.45) | 2.39 (2.35 to 2.42) | 94 |
| Prostate | male | 1.38 (1.36 to 1.41) | 0.48 (0.47 to 0.50) | 1.52 (1.49 to 1.54) | 1.55 (1.52 to 1.57) | 89 |
| Blood | female | 0.55 (0.53 to 0.56) | 0.24 (0.23 to 0.25) | 0.59 (0.57 to 0.61) | 0.59 (0.58 to 0.61) | 92 |
| | male | 0.78 (0.76 to 0.80) | 0.36 (0.34 to 0.37) | 0.84 (0.82 to 0.86) | 0.84 (0.82 to 0.86) | 92 |
| | total | 0.66 (0.65 to 0.68) | 0.30 (0.29 to 0.31) | 0.72 (0.70 to 0.73) | 0.72 (0.71 to 0.73) | 92 |
| Uterine | female | 0.40 (0.38 to 0.41) | 0.13 (0.12 to 0.14) | 0.54 (0.52 to 0.55) | 0.54 (0.53 to 0.56) | 73 |
| Cervical | female | 0.11 (0.11 to 0.12) | 0.05 (0.05 to 0.05) | 0.15 (0.14 to 0.15) | 0.16 (0.15 to 0.16) | 73 |
| Oral | female | 0.07 (0.06 to 0.07) | 0.05 (0.04 to 0.05) | 0.12 (0.11 to 0.13) | 0.12 (0.12 to 0.13) | 52 |
| | male | 0.16 (0.15 to 0.17) | 0.12 (0.11 to 0.12) | 0.27 (0.26 to 0.28) | 0.28 (0.26 to 0.29) | 59 |
| | total | 0.11 (0.11 to 0.12) | 0.08 (0.08 to 0.09) | 0.20 (0.19 to 0.20) | 0.20 (0.19 to 0.21) | 57 |
| Testis | male | 0.12 (0.11 to 0.12) | 0.00 (0.00 to 0.01) | 0.15 (0.14 to 0.16) | 0.15 (0.15 to 0.16) | 75 |
| Liver | female | 0.02 (0.02 to 0.03) | 0.10 (0.09 to 0.10) | 0.11 (0.11 to 0.12) | 0.12 (0.11 to 0.12) | 21 |
| | male | 0.08 (0.08 to 0.09) | 0.17 (0.17 to 0.18) | 0.19 (0.18 to 0.20) | 0.19 (0.18 to 0.20) | 42 |
| | total | 0.05 (0.05 to 0.06) | 0.14 (0.13 to 0.14) | 0.15 (0.15 to 0.16) | 0.15 (0.15 to 0.16) | 34 |
| Any cancer | female | 5.63 (5.58 to 5.68) | 3.00 (2.97 to 3.04) | 7.16 (7.11 to 7.22) | 7.52 (7.46 to 7.57) | 76 |
| | male | 5.56 (5.51 to 5.61) | 3.72 (3.68 to 3.76) | 7.14 (7.08 to 7.20) | 7.49 (7.43 to 7.55) | 76 |
| | total | 5.59 (5.56 to 5.63) | 3.36 (3.34 to 3.39) | 7.15 (7.11 to 7.19) | 7.50 (7.46 to 7.54) | 76 |

**Supplementary Table 4. Crude incidence rates of cancer types per 1000 person years (95% CI) in QResearch and CPRD based on GP data only (as linked data unavailable for CPRD).**

| *outcome* | | *QResearch GP record only* | *CPRD GP record only* |
|---|---|---|---|
| Lung | female | 0.60 (0.58 to 0.62) | 0.68 (0.65 to 0.71) |
| | male | 0.71 (0.69 to 0.73) | 0.77 (0.74 to 0.80) |
| | total | 0.65 (0.64 to 0.67) | 0.72 (0.70 to 0.75) |
| Colorectal | female | 0.54 (0.53 to 0.56) | 0.60 (0.58 to 0.63) |
| | male | 0.70 (0.69 to 0.72) | 0.75 (0.72 to 0.79) |
| | total | 0.62 (0.61 to 0.63) | 0.68 (0.66 to 0.70) |
| Gastro-oesophageal | female | 0.17 (0.16 to 0.18) | 0.20 (0.18 to 0.22) |
| | male | 0.42 (0.41 to 0.43) | 0.47 (0.45 to 0.50) |
| | total | 0.30 (0.29 to 0.30) | 0.34 (0.32 to 0.35) |
| Pancreas | female | 0.16 (0.15 to 0.17) | 0.16 (0.15 to 0.18) |
| | male | 0.19 (0.18 to 0.20) | 0.17 (0.16 to 0.19) |
| | total | 0.18 (0.17 to 0.18) | 0.17 (0.15 to 0.18) |
| Ovarian | female | 0.24 (0.23 to 0.25) | 0.26 (0.24 to 0.28) |
| Renal | female | 0.29 (0.27 to 0.30) | 0.26 (0.24 to 0.28) |
| | male | 0.76 (0.74 to 0.78) | 0.65 (0.62 to 0.68) |
| | total | 0.53 (0.52 to 0.54) | 0.46 (0.44 to 0.47) |
| Breast | female | 2.24 (2.21 to 2.27) | 2.17 (2.12 to 2.23) |
| Prostate | male | 1.38 (1.36 to 1.41) | 1.29 (1.24 to 1.33) |
| Blood | female | 0.55 (0.53 to 0.56) | 0.54 (0.51 to 0.56) |
| | male | 0.78 (0.76 to 0.80) | 0.76 (0.73 to 0.79) |
| | total | 0.66 (0.65 to 0.68) | 0.65 (0.63 to 0.67) |
| Uterine | female | 0.40 (0.38 to 0.41) | 0.42 (0.40 to 0.44) |
| Cervical | female | 0.11 (0.11 to 0.12) | 0.13 (0.12 to 0.15) |
| Oral | female | 0.07 (0.06 to 0.07) | 0.09 (0.08 to 0.10) |
| | male | 0.16 (0.15 to 0.17) | 0.20 (0.18 to 0.22) |
| | total | 0.11 (0.11 to 0.12) | 0.14 (0.13 to 0.15) |
| Testis | male | 0.12 (0.11 to 0.12) | 0.11 (0.10 to 0.13) |
| Liver | female | 0.02 (0.02 to 0.03) | 0.03 (0.02 to 0.03) |
| | male | 0.08 (0.08 to 0.09) | 0.09 (0.08 to 0.10) |
| | total | 0.05 (0.05 to 0.06) | 0.06 (0.05 to 0.06) |
| **Any cancer** | **female** | **5.63 (5.58 to 5.68)** | **5.71 (5.62 to 5.81)** |
| | **male** | **5.56 (5.51 to 5.61)** | **5.36 (5.27 to 5.45)** |
| | **total** | **5.59 (5.56 to 5.63)** | **5.54 (5.47 to 5.60)** |

**Supplementary Table 5 Candidate predictor variables evaluated for inclusion in new models compared with existing predictors in QCancer.**

| | **Predictors included in final models A or B** | | **Not included in final models A or B** |
|---|---|---|---|
| | ***Existing predictors included in existing QCancer models*** | ***New candidate predictors tested and included in final models*** | ***New candidate predictors tested but not included in final models*** |
| **Factors on or prior to cohort entry** | Age, body mass index, smoking status, alcohol use, Townsend deprivation score.<br><br>Chronic obstructive pulmonary disease, chronic pancreatitis, type 2 diabetes | Liver cirrhosis, hepatitis C, hepatitis B, HIV/AIDS. | Endometriosis, polycystic ovarian disease, rheumatoid arthritis, systemic lupus erythematosus, primary biliary cirrhosis, inflammatory bowel disease, benign breast disease |
| **Family History recorded prior to cohort entry** | breast cancer<br>gastro-intestinal cancer<br>prostate cancer<br>ovarian cancer | lung cancer<br>blood cancer | n/a |
| **Red Flag symptoms** | Thrombosis, abdominal distension, abdominal pain, appetite loss, heartburn, indigestion, dysphagia, haematemesis, rectal bleeding, haematuria, haemoptysis, neck lump, weight loss, night sweats, breast lump, breast pain, nipple discharge or breast skin changes, inter-menstrual bleeding, post-menopausal bleeding, post-coital bleeding, testicular lump, testicular pain. | No new ones tested. | n/a |
| **Non-specific or vague symptoms in the 12 months prior to cohort entry** | Change in bowel habit, bruising, cough, urinary retention, nocturia, urinary frequency | back pain, hoarseness, flatulence, abdominal mass, dark urine | Itching, bruising, Nausea, flushes, thirst, diarrhoea, pelvic pain, shoulder pain, tiredness, vaginal discharge, headache (jaundice and steatorrhea not included as too few events) |
| **Most recent blood tests in the two years prior to cohort entry** | Haemoglobin as a binary variable (<110 g/L). | Haemoglobin, platelets, bilirubin, neutrophil, lymphocytes, albumin, alkaline phosphatase. | Glycosylated haemoglobin, alanine transaminase, HBA1c random blood glucose, creatinine, alanine transaminase. |

**Supplementary Table 6 Summary of associations between probability of cancer and individual blood test results. Decreased means there is an increased probability of cancer associated with lower values; increased means there is an increased probability of cancer associated with higher values; no association means no association was found between cancer probability and the value**

| | | ***Haemoglobin*** | ***Neutrophils*** | ***Lymphocytes*** | ***Platelets*** | ***Albumin*** | ***Alkaline Phosphatase*** | ***Bilirubin*** |
|---|---|---|---|---|---|---|---|---|
| **Men** | Lung | Decreased | Increased | Decreased | Increased | Decreased | Increased | No association |
| | Colorectal | Decreased | Increased | Decreased | Increased | Decreased | Increased | No association |
| | Prostate | Increased | Decreased | Increased | Increased | Increased | No association | No association |
| | Blood | Decreased | Decreased | Increased | No association | Decreased | Increased | Increased |
| | Renal | No association | Increased | Decreased | Increased | Increased | No association | No association |
| | Gastro-oesophageal | Decreased | Increased | Decreased | Increased | No association | Increased | No association |
| | Pancreas | No association | Increased | Decreased | No association | No association | Increased | Increased |
| | Testicular | No association | No association | Decreased | No association | No association | No association | No association |
| | Oral | No association | Increased | No association | No association | No association | No association | No association |
| | liver | No association | Decreased | Decreased | No association | Decreased | Increased | Increased |
| | Other | No association | No association | Decreased | Increased | No association | No association | No association |
| **Women** | Lung | No association | Increased | No association | Increased | Decreased | Increased | No association |
| | Colorectal | Decreased | Increased | Decreased | Increased | Decreased | Increased | Decreased |
| | Breast | No association | Increased | No association | No association | No association | Decreased | Increased |
| | Blood | Decreased | Decreased | Increased | Increased | Decreased | Increased | Increased |
| | Ovarian | No association | Increased | Decreased | Increased | Decreased | No association | No association |
| | Renal | No association | Increased | Decreased | Increased | No association | No association | No association |
| | Gastro-oesophageal | Decreased | Increased | No association | No association | Decreased | No association | No association |
| | Uterine | No association | Increased | No association | Increased | No association | No association | No association |
| | Pancreas | No association | Increased | No association | No association | No association | Increased | Increased |
| | Cervical | No association | Increased | No association | Increased | Decreased | No association | No association |
| | Oral | No association | No association | No association | No association | No association | Increased | No association |
| | Liver | Decreased | No association | Decreased | No association | Decreased | Increased | Increased |
| | Other | No association | No association | Decreased | Increased | No association | Increased | Increased |

**Supplementary Table 7: Cancer stage at diagnosis in the QResearch English validation cohort restricted to 2015-2020 due to availability of linked cancer registry data. Stages 1 and 2 considered 'early' whilst stages 3 and 4 considered late.**

| | | *Stage 1* | | *Stage 2* | | *Stage 3* | | *Stage 4* | | *Not recorded* | | Total | Stage1 or 2 | |
|---|---|---|---|---|---|---|---|---|---|---|---|---|---|---|
| | | **Count** | **Row %** | **Count** | **Row %** | **Count** | **Row %** | **Count** | **Row %** | **Count** | **Row %** | **Count** | **count** | **Row %** |
| **Women** | lung | 240 | 16.0 | 88 | 5.9 | 253 | 16.9 | 763 | 51.0 | 152 | 10.2 | **1,496** | 328 | 21.9 |
| | colorectal | 208 | 15.1 | 319 | 23.1 | 357 | 25.9 | 363 | 26.3 | 131 | 9.5 | **1,378** | 527 | 38.2 |
| | breast | 1,675 | 34.7 | 1,990 | 41.2 | 485 | 10.0 | 224 | 4.6 | 457 | 9.5 | **4,831** | 3665 | 75.9 |
| | blood | 93 | 8.9 | 104 | 9.9 | 139 | 13.3 | 201 | 19.2 | 511 | 48.8 | **1,048** | 197 | 18.8 |
| | ovarian | 154 | 26.4 | 40 | 6.8 | 146 | 25.0 | 111 | 19.0 | 133 | 22.8 | **584** | 194 | 33.2 |
| | renal | 207 | 35.2 | 81 | 13.8 | 81 | 13.8 | 112 | 19.0 | 107 | 18.2 | **588** | 288 | 49.0 |
| | gastric | 48 | 10.2 | 47 | 10.0 | 122 | 25.9 | 164 | 34.8 | 90 | 19.1 | **471** | 95 | 20.2 |
| | uterine | 633 | 62.2 | 68 | 6.7 | 121 | 11.9 | 80 | 7.9 | 115 | 11.3 | **1,017** | 701 | 68.9 |
| | pancreatic | 23 | 4.9 | 62 | 13.3 | 47 | 10.1 | 241 | 51.6 | 94 | 20.1 | **467** | 85 | 18.2 |
| | cervical | 147 | 50.0 | 46 | 15.6 | 25 | 8.5 | 36 | 12.2 | 40 | 13.6 | **294** | 193 | 65.6 |
| | oral | 48 | 21.5 | 26 | 11.7 | 36 | 16.1 | 90 | 40.4 | 23 | 10.3 | **223** | 74 | 33.2 |
| | liver | 11 | 6.3 | 12 | 6.8 | 10 | 5.7 | 73 | 41.5 | 70 | 39.8 | **176** | 23 | 13.1 |
| | other | 567 | 37.2 | 196 | 12.9 | 123 | 8.1 | 137 | 9.0 | 501 | 32.9 | **1,524** | 763 | 50.1 |
| | ***Total*** | ***4,054*** | ***28.8*** | ***3,079*** | ***21.8*** | ***1,945*** | ***13.8*** | ***2,595*** | ***18.4*** | ***2,424*** | ***17.2*** | **14,097** | 7133 | 50.6 |
| | | | | | | | | | | | | | | |
| **Men** | lung | 219 | 11.7 | 132 | 7.1 | 364 | 19.5 | 946 | 50.6 | 208 | 11.1 | **1,869** | 351 | 18.8 |
| | colorectal | 318 | 16.8 | 372 | 19.6 | 549 | 28.9 | 466 | 24.6 | 192 | 10.1 | **1,897** | 690 | 36.4 |
| | prostate | 1,089 | 33.4 | 507 | 15.5 | 640 | 19.6 | 638 | 19.6 | 387 | 11.9 | **3,261** | 1596 | 48.9 |
| | blood | 131 | 9.3 | 112 | 7.9 | 147 | 10.4 | 304 | 21.6 | 716 | 50.8 | **1,410** | 243 | 17.2 |
| | renal | 592 | 40.8 | 223 | 15.4 | 198 | 13.6 | 237 | 16.3 | 202 | 13.9 | **1,452** | 815 | 56.1 |
| | gastric | 75 | 6.8 | 121 | 11.0 | 294 | 26.8 | 447 | 40.7 | 162 | 14.7 | **1,099** | 196 | 17.8 |
| | pancreatic | 24 | 4.8 | 50 | 10.0 | 49 | 9.8 | 278 | 55.6 | 99 | 19.8 | **500** | 74 | 14.8 |
| | oral | 77 | 14.3 | 69 | 12.8 | 68 | 12.6 | 283 | 52.4 | 43 | 8.0 | **540** | 146 | 27.0 |
| | testicular | 232 | 67.6 | 18 | 5.2 | 21 | 6.1 | 5 | 1.5 | 67 | 19.5 | **343** | 250 | 72.9 |
| | liver | 34 | 9.0 | 43 | 11.4 | 33 | 8.7 | 94 | 24.9 | 174 | 46.0 | **378** | 77 | 20.4 |
| | other | 513 | 32.0 | 218 | 13.6 | 141 | 8.8 | 151 | 9.4 | 582 | 36.3 | **1,605** | 731 | 45.5 |
| | ***Total*** | ***3,304*** | ***23.0*** | ***1,865*** | ***13.0*** | ***2,504*** | ***17.4*** | ***3,849*** | ***26.8*** | ***2,832*** | ***19.7*** | **14,354** | 5169 | 36.0 |

**Supplementary Table 8. Discrimination using c statistic values (95% CI) for early cancer diagnoses (stage 1 and 2) using models A and B (in people aged 18-84) and QCancer (in people aged 25-84) in the QResearch English validation cohort. Analyses restricted from 2015-2020 to match period for which cancer stage data was recorded.**

| | ***Women Model A*** | ***Women Model B*** | ***Women QCancer*** | ***Men Model A*** | ***Men Model B*** | ***Men QCancer*** |
|---|---|---|---|---|---|---|
| any cancer | 0.837 (0.832 to 0.841) | 0.837 (0.833 to 0.842) | 0.849 (0.844 to 0.853) | 0.864 (0.860 to 0.868) | 0.865 (0.860 to 0.869) | 0.816 (0.811 to 0.821) |
| lung | 0.940 (0.930 to 0.950) | 0.943 (0.933 to 0.952) | 0.938 (0.927 to 0.949) | 0.943 (0.932 to 0.954) | 0.945 (0.934 to 0.956) | 0.926 (0.915 to 0.938) |
| colorectal | 0.893 (0.881 to 0.905) | 0.896 (0.883 to 0.909) | 0.893 (0.883 to 0.903) | 0.906 (0.897 to 0.914) | 0.910 (0.901 to 0.919) | 0.873 (0.860 to 0.887) |
| breast | 0.878 (0.873 to 0.884) | 0.880 (0.875 to 0.885) | n/a | n/a | n/a | 0.850 (0.843 to 0.856) |
| prostate | n/a | n/a | 0.858 (0.852 to 0.865) | 0.884 (0.879 to 0.889) | 0.885 (0.879 to 0.890) | n/a |
| blood | 0.762 (0.725 to 0.798) | 0.766 (0.730 to 0.803) | 0.796 (0.766 to 0.827) | 0.820 (0.791 to 0.848) | 0.824 (0.795 to 0.854) | 0.739 (0.704 to 0.774) |
| ovarian | 0.751 (0.712 to 0.789) | 0.767 (0.730 to 0.805) | n/a | n/a | n/a | 0.756 (0.718 to 0.793) |
| renal | 0.869 (0.846 to 0.892) | 0.868 (0.843 to 0.892) | 0.910 (0.899 to 0.921) | 0.930 (0.921 to 0.940) | 0.929 (0.919 to 0.939) | 0.826 (0.798 to 0.853) |
| gastro | 0.909 (0.873 to 0.946) | 0.912 (0.876 to 0.947) | 0.925 (0.905 to 0.945) | 0.933 (0.915 to 0.951) | 0.934 (0.915 to 0.952) | 0.898 (0.857 to 0.939) |
| uterine | 0.919 (0.906 to 0.932) | 0.918 (0.905 to 0.931) | n/a | n/a | n/a | 0.884 (0.867 to 0.900) |
| pancreatic | 0.884 (0.847 to 0.920) | 0.890 (0.853 to 0.928) | 0.865 (0.822 to 0.908) | 0.884 (0.845 to 0.924) | 0.877 (0.833 to 0.920) | 0.852 (0.807 to 0.897) |
| cervical | 0.627 (0.590 to 0.663) | 0.635 (0.597 to 0.674) | n/a | n/a | n/a | 0.611 (0.578 to 0.645) |
| oral | 0.698 (0.636 to 0.760) | 0.701 (0.634 to 0.768) | n/a | 0.809 (0.770 to 0.847) | 0.812 (0.776 to 0.847) | n/a |
| testicular | n/a | n/a | 0.850 (0.820 to 0.879) | 0.847 (0.819 to 0.876) | 0.846 (0.817 to 0.874) | n/a |
| liver | 0.799 (0.696 to 0.902) | 0.862 (0.762 to 0.963) | n/a | 0.911 (0.879 to 0.943) | 0.924 (0.890 to 0.958) | n/a |
| other | 0.700 (0.681 to 0.718) | 0.695 (0.677 to 0.714) | 0.747 (0.731 to 0.763) | 0.777 (0.762 to 0.792) | 0.778 (0.763 to 0.793) | 0.674 (0.655 to 0.692) |

**Supplementary Table 9. Discrimination using c statistic values (95% CI) by cancer type using QCancer in patients aged 25-84 years in the QResearch English validation cohort and CPRD cohorts.**

| | *QResearch cohort* | | *CPRD cohort* | |
|---|---|---|---|---|
| | **QCancer Women** | **QCancer Men** | **QCancer Women** | **QCancer Men** |
| Any cancer | 0.819 (0.817 to 0.822) | 0.859 (0.857 to 0.861) | 0.817 (0.814 to 0.820) | 0.852 (0.849 to 0.854) |
| Lung | 0.908 (0.901 to 0.914) | 0.919 (0.914 to 0.923) | 0.912 (0.906 to 0.918) | 0.916 (0.911 to 0.921) |
| Colorectal | 0.856 (0.848 to 0.864) | 0.874 (0.867 to 0.880) | 0.845 (0.836 to 0.855) | 0.862 (0.854 to 0.869) |
| Breast | 0.842 (0.838 to 0.847) | n/a | 0.823 (0.817 to 0.829) | |
| Prostate | n/a | 0.872 (0.869 to 0.876) | | 0.860 (0.855 to 0.864) |
| Blood | 0.768 (0.757 to 0.779) | 0.792 (0.783 to 0.801) | 0.759 (0.746 to 0.772) | 0.793 (0.784 to 0.802) |
| Ovarian | 0.791 (0.776 to 0.807) | n/a | 0.807 (0.789 to 0.824) | |
| Renal | 0.858 (0.845 to 0.870) | 0.912 (0.906 to 0.917) | 0.832 (0.815 to 0.849) | 0.891 (0.882 to 0.899) |
| Gastro-oesophageal | 0.882 (0.867 to 0.897) | 0.921 (0.914 to 0.928) | 0.873 (0.856 to 0.889) | 0.915 (0.907 to 0.923) |
| Uterine | 0.858 (0.847 to 0.870) | n/a | 0.867 (0.852 to 0.881) | |
| Pancreas | 0.843 (0.827 to 0.860) | 0.873 (0.859 to 0.887) | 0.847 (0.826 to 0.867) | 0.872 (0.854 to 0.889) |
| Cervical | 0.667 (0.644 to 0.691) | n/a | 0.672 (0.646 to 0.698) | |
| Testicular | n/a | 0.834 (0.812 to 0.856) | | 0.783 (0.754 to 0.813) |
| Other | 0.699 (0.689 to 0.709) | 0.754 (0.746 to 0.763) | 0.721 (0.710 to 0.733) | 0.767 (0.757 to 0.777) |

**Supplementary Table 10. Discrimination using polytomous discrimination index (PDI) estimates overall and by cancer type using Model A and B in men and women aged 18-84 years in the QResearch English validation cohort and CPRD cohorts.**

| | | ***QResearch*** | ***QResearch*** | ***CPRD*** | ***CPRD*** |
|---|---|---|---|---|---|
| | | **Model A** | **Model B** | **Model A** | **Model B** |
| Women | | **PDI** | **PDI** | **PDI** | **PDI** |
| | Overall | 0.257 | 0.266 | 0.258 | 0.272 |
| | | | | | |
| | Any cancer | 0.387 | 0.392 | 0.377 | 0.382 |
| | Lung | 0.279 | 0.275 | 0.277 | 0.274 |
| | Colorectal | 0.232 | 0.242 | 0.227 | 0.245 |
| | Breast | 0.428 | 0.429 | 0.422 | 0.423 |
| | Blood | 0.129 | 0.151 | 0.102 | 0.139 |
| | Ovarian | 0.149 | 0.163 | 0.162 | 0.194 |
| | Renal tract | 0.397 | 0.396 | 0.362 | 0.365 |
| | Gastro | 0.355 | 0.357 | 0.356 | 0.352 |
| | Uterine | 0.440 | 0.439 | 0.443 | 0.442 |
| | Pancreatic | 0.192 | 0.205 | 0.2 | 0.226 |
| | Cervical | 0.159 | 0.159 | 0.167 | 0.179 |
| | Oral | 0.221 | 0.220 | 0.227 | 0.226 |
| | Liver | 0.171 | 0.242 | 0.228 | 0.298 |
| | | | | | |
| Men | Overall | 0.308 | 0.323 | 0.295 | 0.31 |
| | | | | | |
| | Any cancer | 0.464 | 0.467 | 0.437 | 0.442 |
| | Lung | 0.292 | 0.301 | 0.276 | 0.283 |
| | Colorectal | 0.261 | 0.275 | 0.239 | 0.249 |
| | Prostate | 0.115 | 0.125 | 0.115 | 0.128 |
| | Blood | 0.129 | 0.159 | 0.104 | 0.136 |
| | Renal tract | 0.5 | 0.503 | 0.466 | 0.47 |
| | Gastro | 0.421 | 0.422 | 0.422 | 0.424 |
| | Pancreatic | 0.248 | 0.25 | 0.274 | 0.269 |
| | Testicular | 0.641 | 0.64 | 0.541 | 0.538 |
| | Oral | 0.331 | 0.331 | 0.277 | 0.277 |
| | Liver | 0.239 | 0.345 | 0.319 | 0.426 |

**Supplementary Table 11. Calibration intercept (CITL) and calibration slope estimates (95% CI) by cancer type for Models A and B in patients aged 25-84 years in the CPRD external cohort.**

| | *Model A women* | *Model B women* | *Model A men* | *Model B men* |
|---|---|---|---|---|
| **CITL** | | | | |
| Lung | -0.64 (-0.69 to -0.59) | -0.62 (-0.67 to -0.57) | -0.60 (-0.64 to -0.55) | -0.58 (-0.62 to -0.53) |
| Colorectal | -0.40 (-0.45 to -0.35) | -0.40 (-0.45 to -0.35) | -0.37 (-0.42 to -0.33) | -0.33 (-0.38 to -0.29) |
| Breast | 0.32 (0.25 to 0.38) | 0.31 (0.24 to 0.37) | n/a | n/a |
| Blood | -0.39 (-0.44 to -0.33) | -0.37 (-0.42 to -0.32) | -0.35 (-0.39 to -0.31) | -0.31 (-0.35 to -0.27) |
| Ovarian | -0.41 (-0.49 to -0.34) | -0.41 (-0.49 to -0.33) | n/a | n/a |
| Renal | -0.63 (-0.71 to -0.56) | -0.63 (-0.71 to -0.56) | -0.65 (-0.70 to -0.60) | -0.66 (-0.71 to -0.61) |
| Gastric | -0.37 (-0.46 to -0.28) | -0.37 (-0.45 to -0.28) | -0.23 (-0.29 to -0.18) | -0.22 (-0.27 to -0.16) |
| Uterine | -0.44 (-0.50 to -0.38) | -0.45 (-0.51 to -0.39) | n/a | n/a |
| Pancreatic | -0.60 (-0.69 to -0.50) | -0.54 (-0.63 to -0.44) | -0.62 (-0.71 to -0.53) | -0.57 (-0.67 to -0.48) |
| Cervical | -0.43 (-0.54 to -0.33) | -0.45 (-0.55 to -0.34) | n/a | n/a |
| Oral | -0.74 (-0.86 to -0.61) | -0.72 (-0.84 to -0.59) | -0.57 (-0.65 to -0.48) | -0.57 (-0.65 to -0.48) |
| Other | -0.55 (-0.59 to -0.50) | -0.54 (-0.59 to -0.50) | -0.56 (-0.61 to -0.52) | -0.56 (-0.60 to -0.52) |
| Liver | -1.77 (-2.00 to -1.54) | -1.67 (-1.90 to -1.44) | -1.00 (-1.13 to -0.88) | -0.92 (-1.04 to -0.79) |
| Prostate | n/a | n/a | -0.52 (-0.56 to -0.49) | -0.58 (-0.61 to -0.54) |
| Testicular | n/a | n/a | -0.23 (-0.34 to -0.11) | -0.21 (-0.32 to -0.09) |
| **Slope** | | | | |
| Lung | 1.08 (1.04 to 1.11) | 1.07 (1.03 to 1.10) | 1.08 (1.04 to 1.11) | 1.08 (1.05 to 1.11) |
| Colorectal | 1.06 (1.03 to 1.10) | 1.06 (1.03 to 1.09) | 1.06 (1.03 to 1.09) | 1.06 (1.04 to 1.09) |
| Breast | 1.13 (1.12 to 1.15) | 1.13 (1.12 to 1.15) | n/a | n/a |
| Blood | 1.05 (1.00 to 1.09) | 1.07 (1.03 to 1.12) | 1.09 (1.05 to 1.12) | 1.08 (1.05 to 1.12) |
| Ovarian | 1.17 (1.12 to 1.23) | 1.16 (1.11 to 1.21) | n/a | n/a |
| Renal | 1.10 (1.06 to 1.13) | 1.10 (1.06 to 1.13) | 1.05 (1.03 to 1.07) | 1.05 (1.03 to 1.07) |
| Gastric | 1.11 (1.07 to 1.16) | 1.11 (1.06 to 1.15) | 1.10 (1.07 to 1.13) | 1.10 (1.07 to 1.13) |
| Uterine | 1.09 (1.06 to 1.11) | 1.09 (1.06 to 1.11) | n/a | n/a |
| Pancreatic | 1.14 (1.08 to 1.21) | 1.13 (1.08 to 1.19) | 1.09 (1.04 to 1.15) | 1.05 (1.00 to 1.11) |
| Cervical | 0.97 (0.91 to 1.03) | 0.97 (0.91 to 1.03) | n/a | n/a |
| Oral | 1.06 (0.99 to 1.13) | 1.06 (0.98 to 1.13) | 1.02 (0.98 to 1.07) | 1.02 (0.98 to 1.06) |
| Other | 1.14 (1.08 to 1.19) | 1.18 (1.13 to 1.23) | 1.15 (1.10 to 1.20) | 1.16 (1.11 to 1.21) |
| Liver | 1.11 (0.97 to 1.25) | 1.03 (0.92 to 1.14) | 1.20 (1.13 to 1.27) | 1.11 (1.05 to 1.16) |
| Prostate | n/a | n/a | 1.20 (1.16 to 1.24) | 1.20 (1.16 to 1.24) |
| Testicular | n/a | n/a | 1.01 (0.97 to 1.06) | 1.02 (0.97 to 1.07) |

**Supplementary Table 12. Calibration intercept (CITL) and calibration slope estimates (95% CI) by cancer type for QCancer in patients aged 25-84 years in the QResearch English validation cohort and CPRD external cohort.**

| | *QResearch cohort* | | *CPRD cohort* | |
|---|---|---|---|---|
| | **QCancer Women** | **QCancer Men** | **QCancer Women** | **QCancer Men** |
| **CITL** | | | | |
| Lung | 0.41 (0.36 to 0.45) | 0.06 (0.02 to 0.10) | -0.17 (-0.22 to -0.12) | -0.50 (-0.55 to -0.46) |
| Colorectal | 0.25 (0.20 to 0.29) | 0.13 (0.09 to 0.17) | -0.17 (-0.22 to -0.12) | -0.35 (-0.40 to -0.31) |
| Breast | -0.39 (-0.44 to -0.34) | n/a | -0.18 (-0.25 to -0.12) | n/a |
| Blood | 0.46 (0.41 to 0.50) | 0.57 (0.53 to 0.61) | 0.09 (0.03 to 0.14) | 0.24 (0.19 to 0.28) |
| Ovarian | -0.16 (-0.22 to -0.09) | n/a | -0.54 (-0.62 to -0.46) | n/a |
| Renal | 0.61 (0.55 to 0.67) | 0.67 (0.64 to 0.71) | 0.10 (0.02 to 0.17) | 0.02 (-0.03 to 0.07) |
| Gastric | -0.02 (-0.10 to 0.05) | -0.24 (-0.29 to -0.19) | -0.36 (-0.45 to -0.27) | -0.46 (-0.52 to -0.41) |
| Uterine | 0.74 (0.69 to 0.79) | n/a | 0.34 (0.28 to 0.40) | n/a |
| Pancreas | 0.48 (0.40 to 0.55) | 0.38 (0.31 to 0.45) | -0.10 (-0.20 to -0.01) | -0.23 (-0.32 to -0.14) |
| Cervical | 0.19 (0.10 to 0.28) | n/a | -0.26 (-0.37 to -0.16) | n/a |
| Other | 0.40 (0.37 to 0.43) | 0.34 (0.31 to 0.37) | -0.24 (-0.28 to -0.20) | -0.27 (-0.31 to -0.24) |
| Prostate | n/a | 0.15 (0.12 to 0.18) | n/a | -0.33 (-0.36 to -0.30) |
| Testis | n/a | -0.51 (-0.61 to -0.41) | n/a | -0.54 (-0.66 to -0.41) |
| | | | | |
| **Slope** | | | | |
| Lung | 0.95 (0.92 to 0.97) | 0.99 (0.96 to 1.01) | 1.03 (1.00 to 1.05) | 1.07 (1.04 to 1.10) |
| Colorectal | 0.93 (0.91 to 0.96) | 0.90 (0.88 to 0.92) | 1.04 (1.00 to 1.07) | 0.97 (0.94 to 1.00) |
| Breast | 0.87 (0.86 to 0.88) | n/a | 0.99 (0.97 to 1.00) | n/a |
| Blood | 0.91 (0.88 to 0.95) | 0.94 (0.91 to 0.97) | 1.06 (1.02 to 1.10) | 1.09 (1.05 to 1.13) |
| Ovarian | 0.82 (0.78 to 0.87) | n/a | 1.02 (0.96 to 1.07) | n/a |
| Renal | 0.92 (0.89 to 0.94) | 0.97 (0.96 to 0.99) | 0.99 (0.96 to 1.03) | 1.02 (1.00 to 1.04) |
| Gastric | 0.92 (0.89 to 0.95) | 0.96 (0.93 to 0.98) | 1.04 (1.00 to 1.08) | 1.06 (1.04 to 1.09) |
| Uterine | 0.94 (0.92 to 0.96) | n/a | 1.03 (1.01 to 1.06) | n/a |
| Pancreas | 0.95 (0.90 to 1.00) | 1.02 (0.97 to 1.06) | 1.12 (1.05 to 1.18) | 1.15 (1.10 to 1.21) |
| Cervical | 0.85 (0.80 to 0.91) | n/a | 0.78 (0.72 to 0.83) | n/a |
| Other | 0.80 (0.77 to 0.83) | 0.90 (0.87 to 0.92) | 0.96 (0.92 to 1.00) | 1.03 (1.00 to 1.07) |
| Prostate | n/a | 0.83 (0.80 to 0.85) | n/a | 0.97 (0.94 to 1.01) |
| Testis | n/a | 0.84 (0.81 to 0.87) | n/a | 0.87 (0.83 to 0.91) |

**Supplementary Table 13. Sensitivity of Model A, Model B and QCancer for identifying cases of cancer by type and stage at diagnosis in men and women in QResearch validation cohort at the 3% threshold. Analyses for early-stage diagnosis were restricted to 2015-2020.**

| | | ***Women*** | | ***Men*** | |
|---|---|---|---|---|---|
| | | **all stages 2015-2023** | **early stage 2015-2020** | **all stages 2015-2023** | **early stage 2015-2020** |
| Any cancer | Total | 22105 | 7133 | 22879 | 5169 |
| | QCancer | 14582 (66.0) | 4509 (63.2) | 17878 (78.1) | 3948 (76.4) |
| | Model A | 17110 (77.4) | 5361 (75.2) | 18903 (82.6) | 4201 (81.3) |
| | Model B | 17024 (77.0) | 5304 (74.4) | 18891 (82.6) | 4180 (80.9) |
| Lung cancer | Total | 2091 | 328 | 2661 | 351 |
| | QCancer | 387 (18.5) | 58 (17.7) | 785 (29.5) | 121 (34.5) |
| | Model A | 697 (33.3) | 110 (33.5) | 980 (36.8) | 149 (42.5) |
| | Model B | 727 (34.8) | 114 (34.8) | 1035 (38.9) | 155 (44.2) |
| Colorectal cancer | Total | 2003 | 527 | 2667 | 690 |
| | QCancer | 369 (18.4) | 102 (19.4) | 678 (25.4) | 194 (28.1) |
| | Model A | 413 (20.6) | 112 (21.3) | 725 (27.2) | 210 (30.4) |
| | Model B | 515 (25.7) | 143 (27.1) | 814 (30.5) | 220 (31.9) |
| Breast cancer | Total | 7158 | 3665 | n/a | n/a |
| | QCancer | 3544 (49.5) | 1817 (49.6) | n/a | n/a |
| | Model A | 3594 (50.2) | 1840 (50.2) | n/a | n/a |
| | Model B | 3592 (50.2) | 1842 (50.3) | n/a | n/a |
| Blood cancer | Total | 1801 | 197 | 2593 | 243 |
| | QCancer | 101 (5.6) | 19 (9.6) | 209 (8.1) | 31 (12.8) |
| | Model A | 149 (8.3) | 24 (12.2) | 289 (11.1) | 42 (17.3) |
| | Model B | 171 (9.5) | 27 (13.7) | 405 (15.6) | 47 (19.3) |
| Ovarian cancer | Total | 872 | 194 | n/a | n/a |
| | QCancer | 55 (6.3) | 8 (4.1) | n/a | n/a |
| | Model A | 48 (5.5) | 7 (3.6) | n/a | n/a |
| | Model B | 66 (7.6) | 9 (4.6) | n/a | n/a |
| Renal cancer | Total | 1190 | 288 | 3189 | 815 |
| | QCancer | 465 (39.1) | 104 (36.1) | 1727 (54.2) | 441 (54.1) |
| | Model A | 487 (40.9) | 106 (36.8) | 1780 (55.8) | 460 (56.4) |
| | Model B | 488 (41.0) | 106 (36.8) | 1779 (55.8) | 460 (56.4) |
| Gastro-oesophageal | Total | 683 | 95 | 1536 | 196 |
| | QCancer | 179 (26.2) | 30 (31.6) | 620 (40.4) | 70 (35.7) |
| | Model A | 174 (25.5) | 30 (31.6) | 606 (39.5) | 66 (33.7) |
| | Model B | 174 (25.5) | 29 (30.5) | 609 (39.6) | 67 (34.2) |
| Uterine cancer | Total | 1541 | 701 | n/a | n/a |
| | QCancer | 670 (43.5) | 356 (50.8) | n/a | n/a |

| | | | | | |
|---|---|---|---|---|---|
| | Model A | 695 (45.1) | 368 (52.5) | n/a | n/a |
| | Model B | 693 (45.0) | 365 (52.1) | n/a | n/a |
| Pancreatic cancer | Total | 675 | 85 | 774 | 74 |
| | QCancer | 19 (2.8) | * | 33 (4.3) | * |
| | Model A | 43 (6.4) | * | 73 (9.4) | * |
| | Model B | 66 (9.8) | * | 115 (14.9) | * |
| Cervical cancer | Total | 459 | 193 | n/a | n/a |
| | QCancer | 8 (1.7) | * | n/a | n/a |
| | Model A | * | * | n/a | n/a |
| | Model B | * | * | n/a | n/a |
| Oral cancer | Total | 373 | 74 | 886 | 146 |
| | Model A | 27 (7.2) | * | 243 (27.4) | 28 (19.2) |
| | Model B | 30 (8.0) | * | 245 (27.7) | 28 (19.2) |
| Liver cancer | Total | 364 | 23 | 601 | 77 |
| | Model A | 12 (3.3) | * | 45 (7.5) | 4 (5.2) |
| | Model B | 34 (9.3) | * | 116 (19.3) | 16 (20.8) |
| Other cancer | Total | 2895 | 763 | 2990 | 731 |
| | QCancer | 97 (3.4) | 18 (2.4) | 174 (5.8) | 26 (3.6) |
| | Model A | 91 (3.1) | 27 (3.5) | 101 (3.4) | 22 (3.0) |
| | Model B | 104 (3.6) | 27 (3.5) | 97 (3.2) | 18 (2.5) |
| Prostate cancer | Total | n/a | n/a | 4500 | 1596 |
| | QCancer | n/a | n/a | 848 (18.8) | 237 (14.8) |
| | Model A | n/a | n/a | 1113 (24.7) | 328 (20.6) |
| | Model B | n/a | n/a | 1214 (27.0) | 361 (22.6) |
| Testicular cancer | Total | n/a | n/a | 482 | 250 |
| | QCancer | n/a | n/a | 247 (51.2) | 137 (54.8) |
| | Model A | n/a | n/a | 165 (34.2) | 93 (37.2) |
| | Model B | n/a | n/a | 147 (30.5) | 79 (31.6) |

*counts suppressed as < 10 events

**Supplementary Table 14. Demographic characteristics of patients reclassified by Model A compared with QCancer in the English validation cohort at the 3% probability threshold. Numbers are counts and column percents.**

| | Women | Women | Women | Women | Men | Men | Men | Men |
|---|---|---|---|---|---|---|---|---|
| **category** | **low on both** | **high QCancer, low model A** | **low QCancer high model A** | **high on both** | **low on both** | **high QCancer, low model A** | **low QCancer high model A** | **high on both** |
| | **no change** | **classified down** | **classified up** | **high on both** | **no change** | **classified down** | **classified up** | **high on both** |
| Total | 1071198 | 3736 | 87446 | 114635 | 1111192 | 8311 | 59103 | 181563 |
| | | | | | | | | |
| <40 years | 577139 (53.9) | 524 (14.0) | 1049 (1.2) | 3656 (3.2) | 615526 (55.4) | 4178 (50.3) | 1258 (2.1) | 598 (0.3) |
| 40-49 years | 188744 (17.6) | 504 (13.5) | 2630 (3.0) | 11729 (10.2) | 234422 (21.1) | 2441 (29.4) | 2255 (3.8) | 1725 (1.0) |
| 50-59 years | 161481 (15.1) | 586 (15.7) | 12049 (13.8) | 18075 (15.8) | 192307 (17.3) | 1204 (14.5) | 7005 (11.9) | 12418 (6.8) |
| 60-69 years | 103142 (9.6) | 1147 (30.7) | 16446 (18.8) | 26644 (23.2) | 68937 (6.2) | 488 (5.9) | 36656 (62.0) | 44041 (24.3) |
| 70+ years | 40692 (3.8) | 975 (26.1) | 55272 (63.2) | 54531 (47.6) | | | 11929 (20.2) | 122781 (67.6) |
| | | | | | | | | |
| White | 548430 (51.2) | 1618 (43.3) | 51637 (59.1) | 69044 (60.2) | 510171 (45.9) | 4527 (54.5) | 34507 (58.4) | 106542 (58.7) |
| Indian | 32094 (3.0) | 243 (6.5) | 1192 (1.4) | 1824 (1.6) | 37943 (3.4) | 129 (1.6) | 1204 (2.0) | 3039 (1.7) |
| Pakistani | 17066 (1.6) | 145 (3.9) | 540 (0.6) | 952 (0.8) | 20810 (1.9) | 73 (0.9) | 565 (1.0) | 1513 (0.8) |
| Bangladeshi | 13873 (1.3) | 109 (2.9) | 374 (0.4) | 768 (0.7) | 17368 (1.6) | 56 (0.7) | 326 (0.6) | 938 (0.5) |
| Other Asian | 24722 (2.3) | 110 (2.9) | 785 (0.9) | 1120 (1.0) | 23370 (2.1) | 82 (1.0) | 743 (1.3) | 1710 (0.9) |
| Caribbean | 12673 (1.2) | 126 (3.4) | 1145 (1.3) | 1500 (1.3) | 11539 (1.0) | 92 (1.1) | 566 (1.0) | 2184 (1.2) |
| Black African | 37313 (3.5) | 194 (5.2) | 963 (1.1) | 1453 (1.3) | 38637 (3.5) | 156 (1.9) | 926 (1.6) | 1640 (0.9) |
| Chinese | 13526 (1.3) | 20 (0.5) | 290 (0.3) | 428 (0.4) | 10969 (1.0) | 26 (0.3) | 278 (0.5) | 531 (0.3) |
| Other | 55042 (5.1) | 168 (4.5) | 1668 (1.9) | 2534 (2.2) | 56214 (5.1) | 272 (3.3) | 1359 (2.3) | 2919 (1.6) |
| Ethnicity missing | 316459 (29.5) | 1003 (26.8) | 28852 (33.0) | 35012 (30.5) | 384171 (34.6) | 2898 (34.9) | 18629 (31.5) | 60547 (33.3) |

**Supplementary Table 15. Summary of SNOMED-CT and ICD-10 Clinical code groups used to identify 15 types of cancer. The contents of each group are published at** https://www.qresearch.org/data/qcode-group-library/

| | ***SNOMED-CT code group ID*** | ***ICD-10 code group ID*** |
|---|---|---|
| Lung | 51 | 2021 |
| Colorectal | 2009 | 2007 |
| Invasive breast | 52 | 1925 |
| Prostate | 56 | 2020 |
| Blood | 58 | 2004 |
| Ovarian | 32 | 2015 |
| Renal tract | 57 | 2027 |
| Gastro-oesophageal | 2013 | 2012 |
| Uterine | 54 | 2016 |
| Pancreatic | 216 | 2014 |
| Cervical | 55 | 2017 |
| Oral, lip, pharyngeal | 222 | 2023 |
| Testicular | 219 | 2018 |
| Liver | 2522 | 2753 |

**Supplementary Table 16 Characteristics of patients with complete data for BMI, alcohol and smoking compared with those with incomplete data for these variables.**

| | *Incomplete data* | *Complete data* |
|---|---|---|
| Total | 4707481 | 2757026 |
| men | 2590342 (55.0) | 1251376 (45.4) |
| mean age (SD) | 41.8 (16.6) | 49.3 (17.9) |
| mean Townsend (SD) | 0.5 (3.1) | 0.5 (3.1) |
| White | 2234514 (47.5) | 1545604 (56.1) |
| Indian | 146199 (3.1) | 96065 (3.5) |
| Pakistani | 91776 (1.9) | 62620 (2.3) |
| Bangladeshi | 47189 (1.0) | 39526 (1.4) |
| Other Asian | 105440 (2.2) | 61101 (2.2) |
| Caribbean | 42348 (0.9) | 36317 (1.3) |
| Black African | 118105 (2.5) | 76162 (2.8) |
| Chinese | 72427 (1.5) | 25142 (0.9) |
| Other | 209835 (4.5) | 115034 (4.2) |
| Ethnicity not recorded | 1639648 (34.8) | 699455 (25.4) |
| FH breast cancer | 130609 (2.8) | 123489 (4.5) |
| FH bowel cancer | 71723 (1.5) | 70817 (2.6) |
| FH ovarian cancer | 8349 (0.2) | 8177 (0.3) |
| FH prostate cancer | 21158 (0.4) | 19008 (0.7) |
| FH lung cancer | 28882 (0.6) | 31111 (1.1) |
| FH blood cancer | 950 (0.0) | 1223 (0.0) |
| chronic pancreatitis | 2048 (0.0) | 3200 (0.1) |
| COPD | 32418 (0.7) | 100211 (3.6) |
| type 2 diabetes | 60729 (1.3) | 302169 (11.0) |
| endometrial hyperplasia or polyp | 10446 (0.2) | 10976 (0.4) |
| liver cirrhosis | 4953 (0.1) | 6697 (0.2) |
| Hepatitis C | 8211 (0.2) | 7049 (0.3) |
| Hepatitis B | 10039 (0.2) | 8239 (0.3) |
| HIV/AIDs | 8144 (0.2) | 5631 (0.2) |
| erectile dysfunction | 14429 (0.3) | 37497 (1.4) |
| constipation | 28305 (0.6) | 39977 (1.5) |
| cough | 159178 (3.4) | 204215 (7.4) |
| urinary frequency | 21527 (0.5) | 25992 (0.9) |
| urinary retention | 2893 (0.1) | 4190 (0.2) |
| nocturia | 4530 (0.1) | 6818 (0.2) |
| itching | 10187 (0.2) | 13902 (0.5) |
| change in bowels | 7642 (0.2) | 11012 (0.4) |
| bruising | 3539 (0.1) | 4841 (0.2) |
| back pain | 193851 (4.1) | 198087 (7.2) |
| hoarseness | 5618 (0.1) | 7557 (0.3) |
| flatulence | 27074 (0.6) | 29575 (1.1) |
| abdominal mass | 4646 (0.1) | 4990 (0.2) |
| dark urine | 2000 (0.0) | 2559 (0.1) |
| appetite loss | 14810 (0.3) | 20891 (0.8) |
| weight loss | 22863 (0.5) | 44082 (1.6) |
| abdominal distension | 28297 (0.6) | 30255 (1.1) |
| abdominal pain | 229683 (4.9) | 194127 (7.0) |

| | | |
|---|---|---|
| dysphagia | 17870 (0.4) | 23639 (0.9) |
| heartburn | 86784 (1.8) | 90049 (3.3) |
| indigestion | 56980 (1.2) | 58312 (2.1) |
| haematemesis | 8430 (0.2) | 9011 (0.3) |
| rectal bleeding | 66142 (1.4) | 58498 (2.1) |
| haematuria | 41816 (0.9) | 49120 (1.8) |
| haemoptysis | 10350 (0.2) | 12054 (0.4) |
| lump in the neck | 21545 (0.5) | 18066 (0.7) |
| thrombosis | 24700 (0.5) | 29179 (1.1) |
| night sweats | 9573 (0.2) | 11706 (0.4) |
| breast lump | 98707 (2.1) | 79715 (2.9) |
| breast pain | 38302 (0.8) | 34769 (1.3) |
| breast skin/nipple changes | 11166 (0.2) | 10645 (0.4) |
| inter-menstrual bleeding | 22210 (0.5) | 20799 (0.8) |
| postmenopausal bleeding | 14673 (0.3) | 16237 (0.6) |
| post coital bleeding | 12087 (0.3) | 10400 (0.4) |
| testicular pain | 22259 (0.5) | 11886 (0.4) |
| testicular lump | 29075 (0.6) | 16902 (0.6) |
| mean BMI (SD) | 25.4 (5.5) | 27.3 (5.8) |
| mean haemoglobin (SD) | 138.3 (15.2) | 137.8 (15.2) |
| mean bilirubin (SD) | 10.2 (6.1) | 10.0 (5.7) |
| mean platelet (SD) | 257.7 (68.7) | 257.3 (70.2) |
| mean neutrophil (SD) | 4.2 (1.9) | 4.3 (1.9) |
| mean lymphocyte (SD) | 2.1 (0.9) | 2.1 (1.0) |
| mean albumin (SD) | 42.8 (4.2) | 42.4 (4.1) |
| mean alkaline phosphatase (SD) | 78.5 (35.6) | 80.3 (35.3) |

**Supplementary Figure 1 Smoothed crude incidence rates of different types of cancer per 10,000 per month 2015-2023 in men and women.**

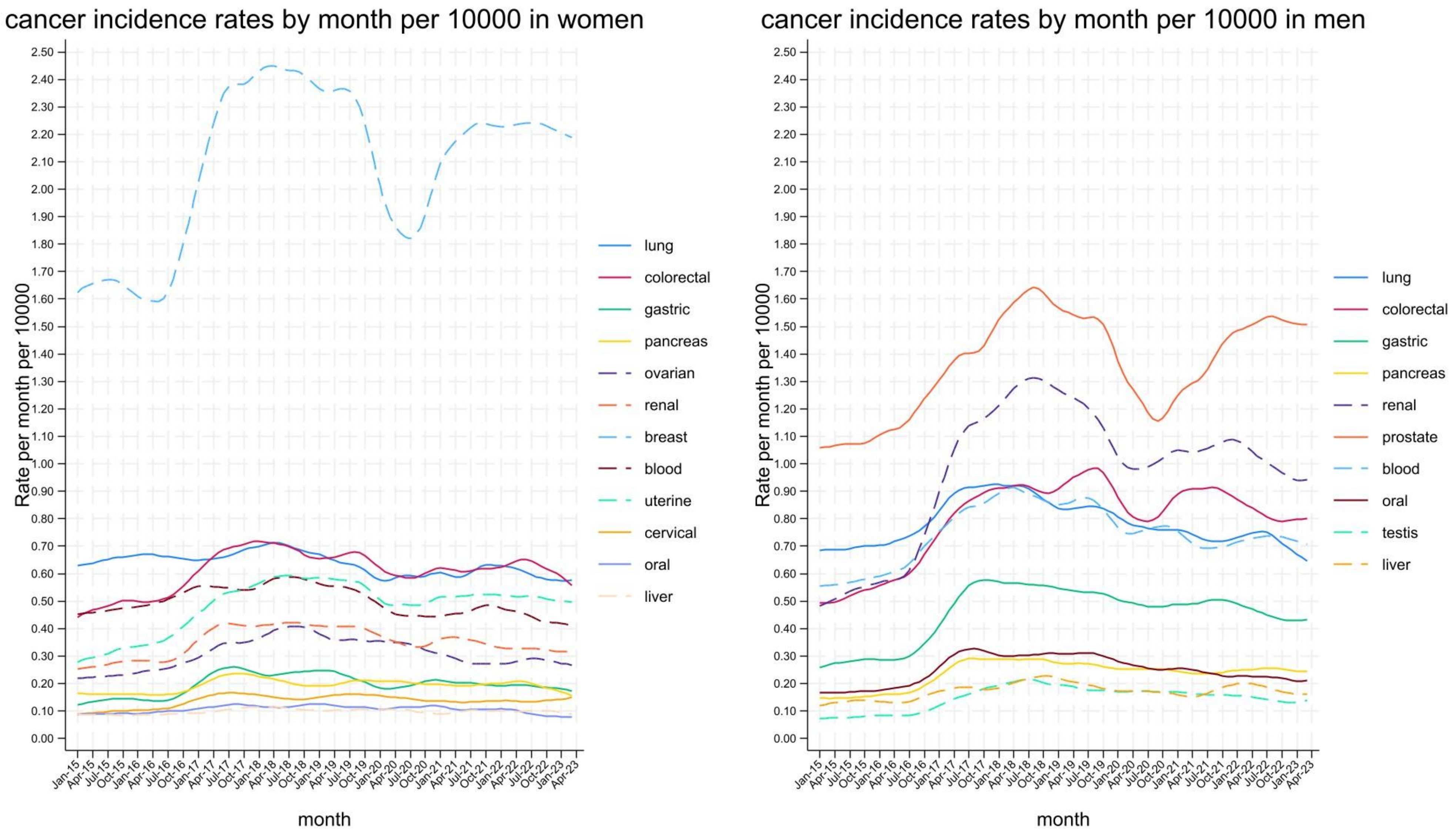

**Supplementary Figure 2. Adjusted Odds ratios (95% CI) for probability of pancreatic, liver, gastro-oesophageal and colorectal cancer for final model B in the QResearch Derivation Cohort (women=red, men=blue). Odds ratios are adjusted for continuous variables (age, BMI, deprivation, blood test results)**

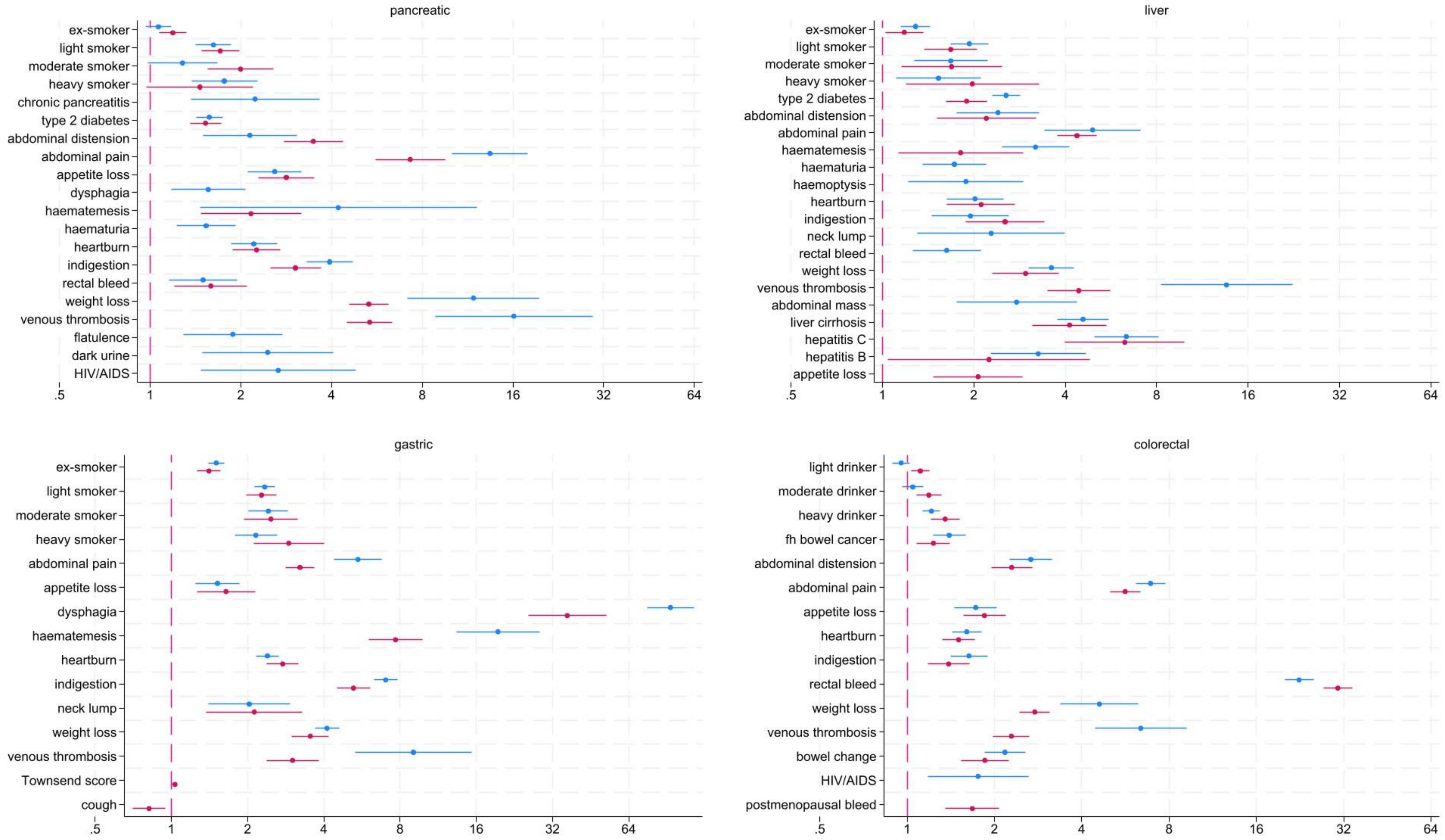

**Supplementary Figure 3. Adjusted Odds ratios (95% CI) for probability of lung, oral, renal and blood cancers for final model B in the Derivation Cohort (women=red, men=blue). Odds ratios are also adjusted for continuous variables (age, BMI, deprivation, blood test results)**

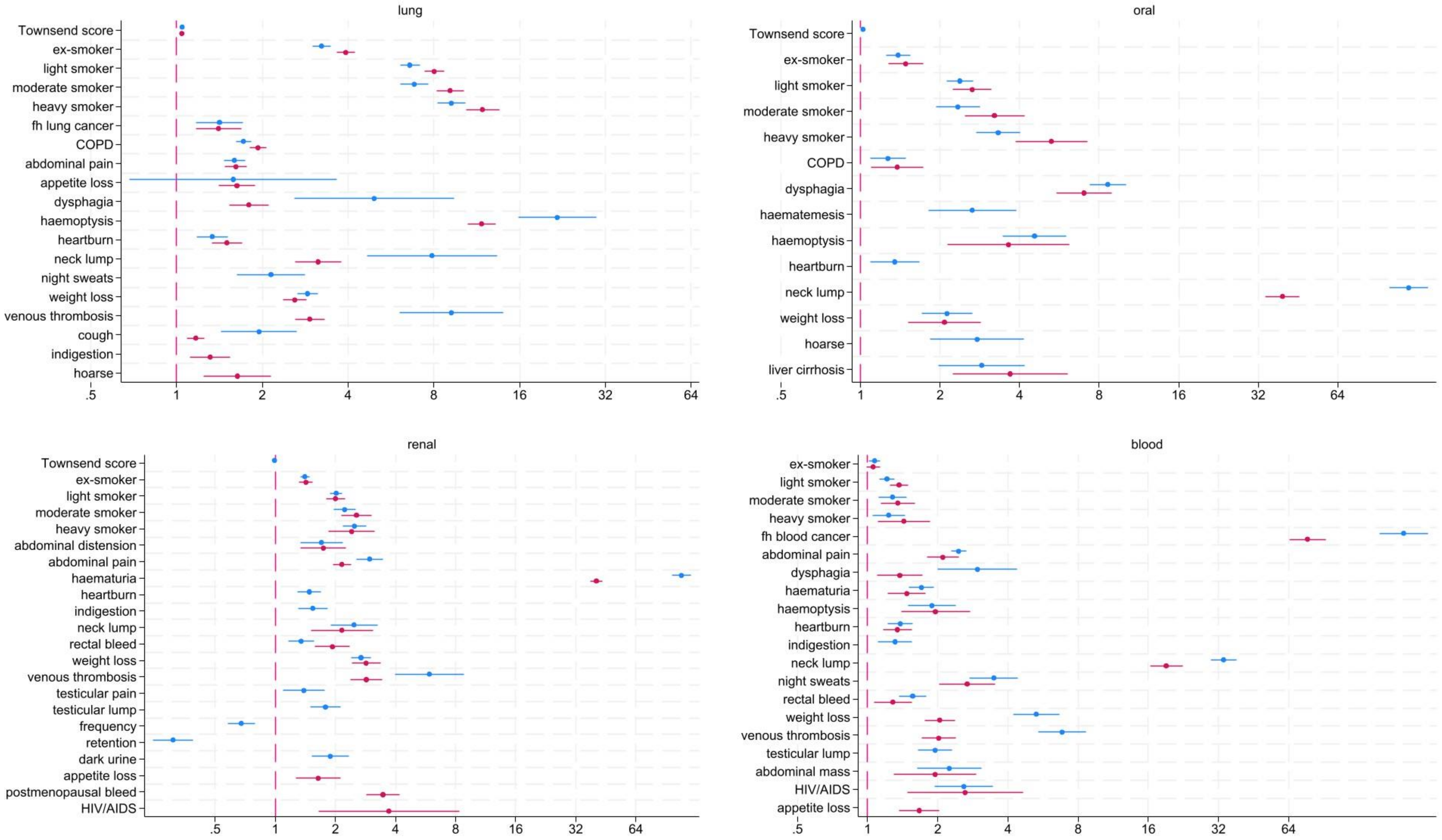

**Supplementary Figure 4. Adjusted Odds ratios (95% CI) for probability for breast, cervical, ovarian, uterine cancers in women for final mod el B in the Derivation Cohort. Odds ratios are also adjusted for continuous variables (age, BMI, deprivation, blood test results)**

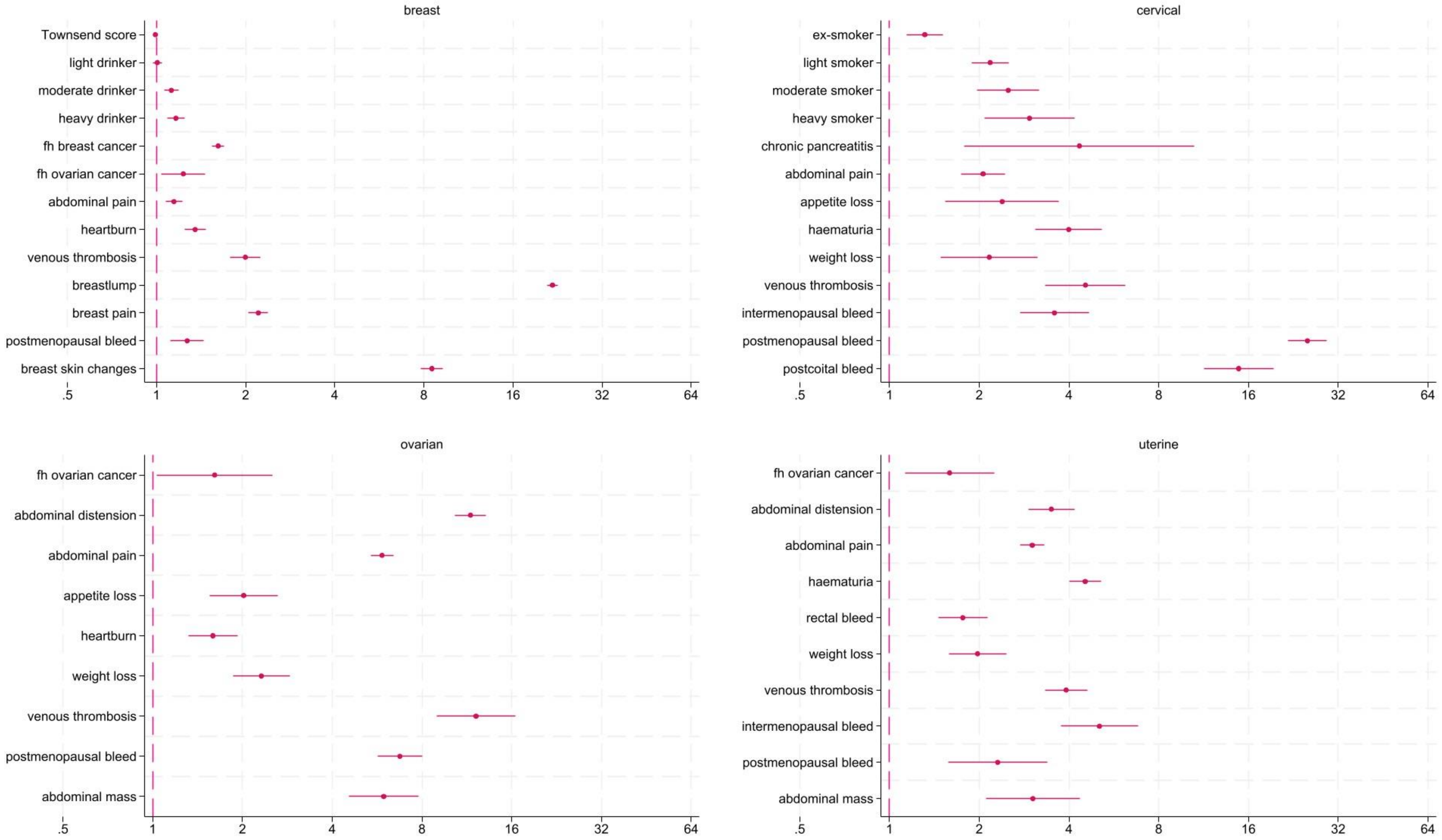

**Supplementary Figure 5. Adjusted Odds ratios (95% CI) for probability of prostate, testicular and other cancers for final model B in the Derivation Cohort (women=red, men=blue)**

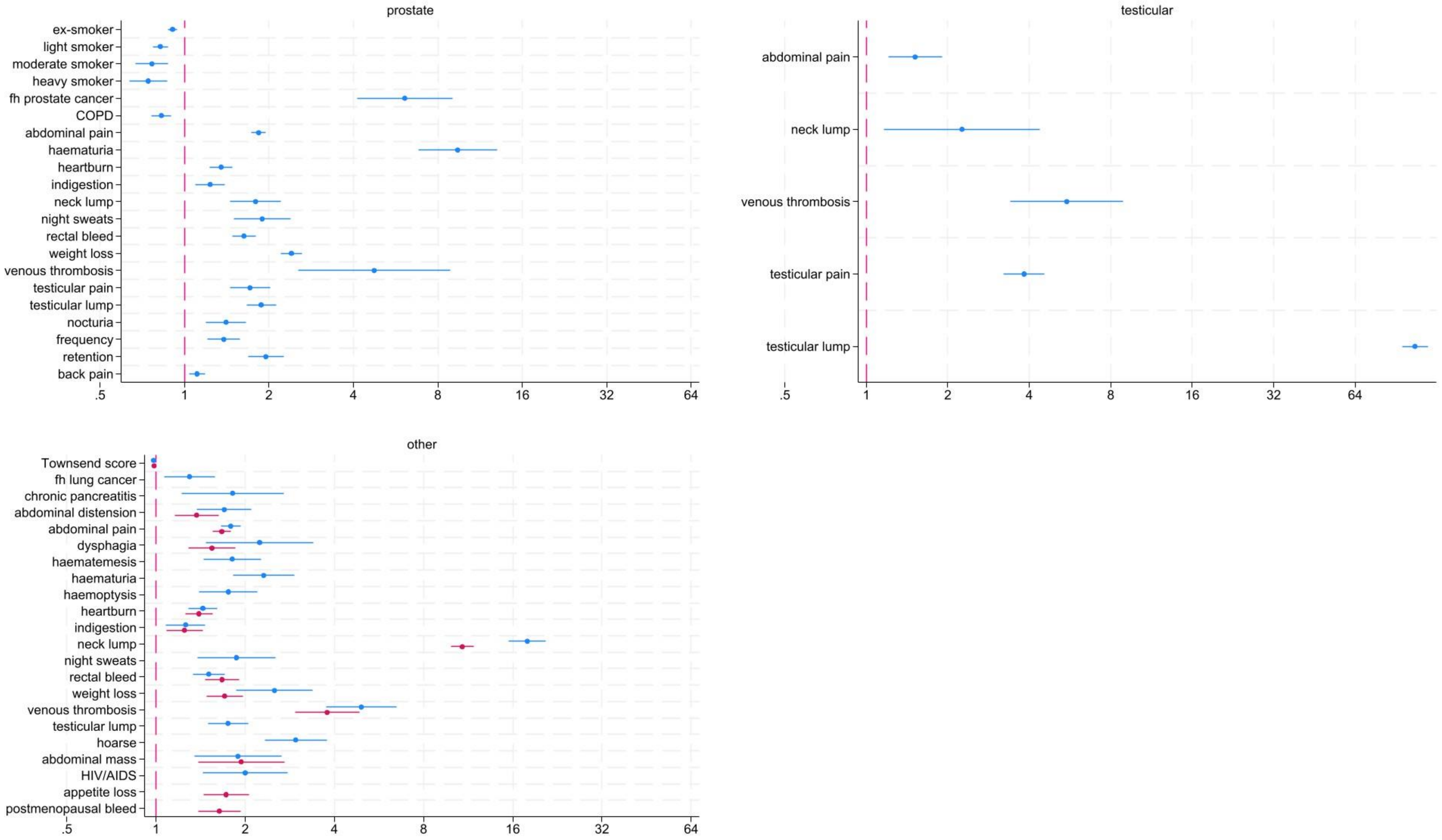

**Supplementary Figure 6. Adjusted odds ratios for probability of each cancer type for symptoms by age in men.**

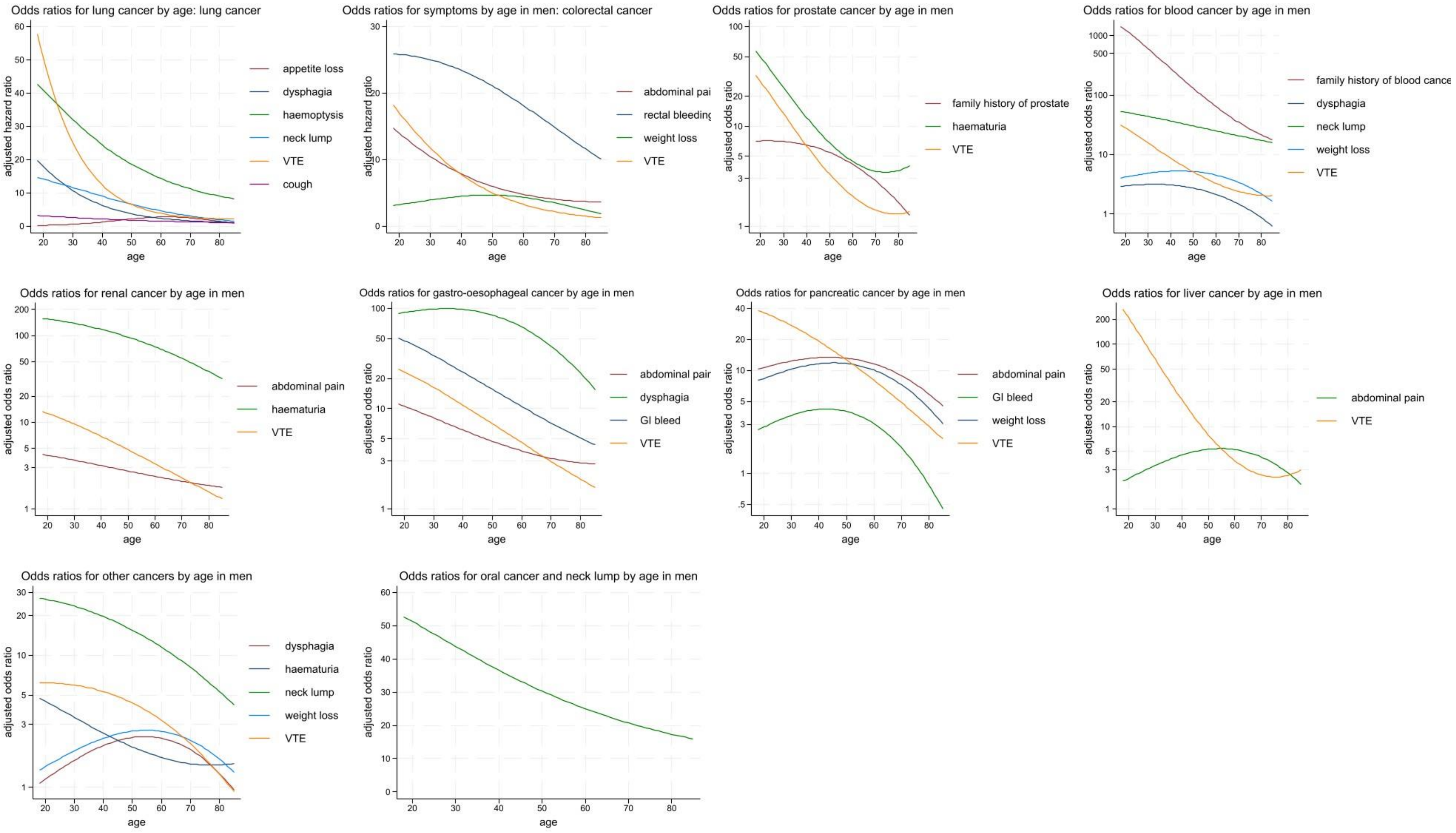

**Supplementary Figure 7. Adjusted odds ratios for probability of each cancer type for symptoms by age in women.**

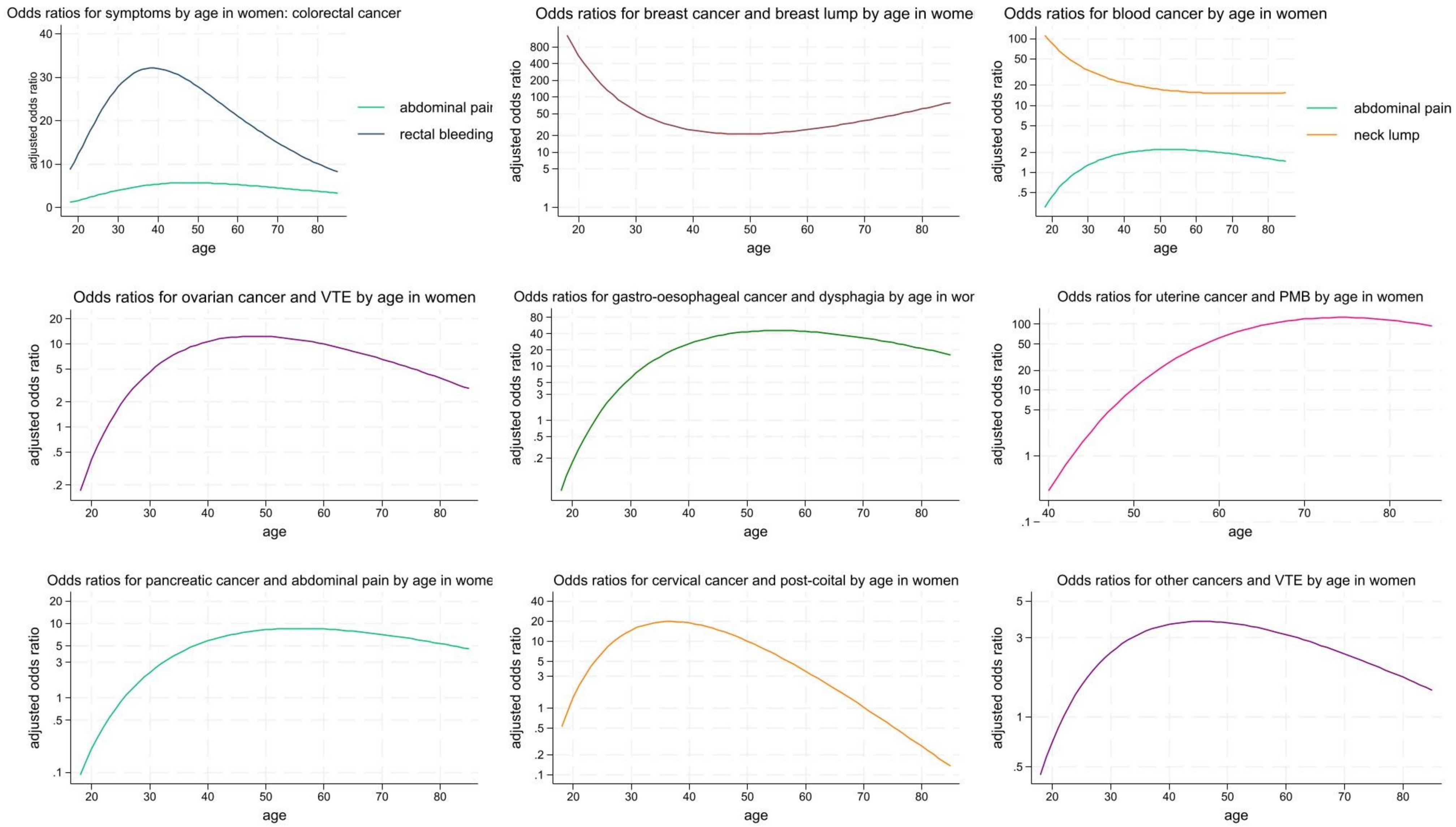

**Supplementary Figure 8. Discrimination values (c statistics) for final model A (without blood tests) in men and women in the QResearch English validation cohort by ethnic group (cells with less than 5 events suppressed).**

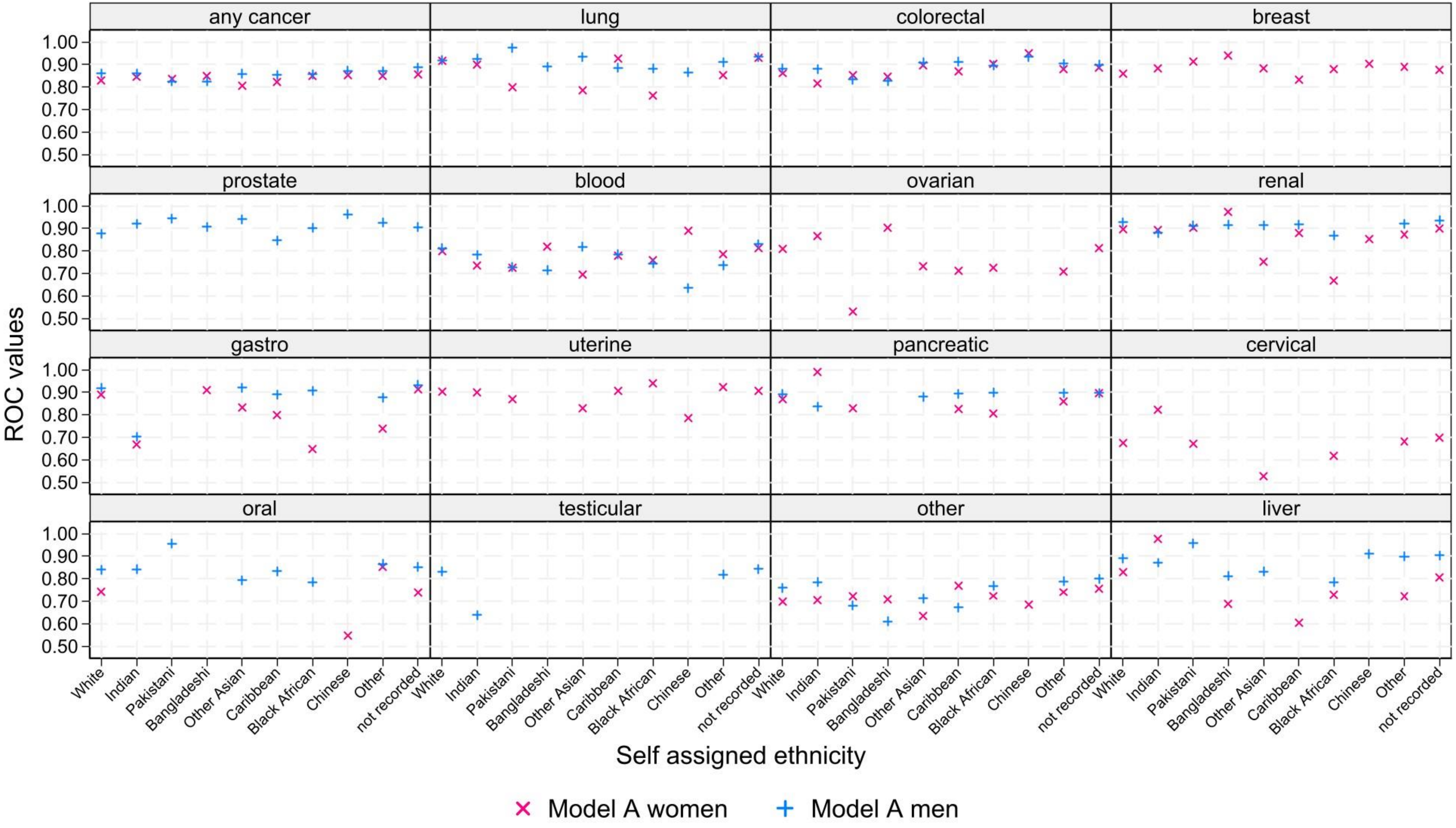

**Supplementary Figure 9. Discrimination values (c statistic) for final model B (with blood tests) in men and women in the QResearch English validation cohort by ethnic group (cells with less than 5 events suppressed).**

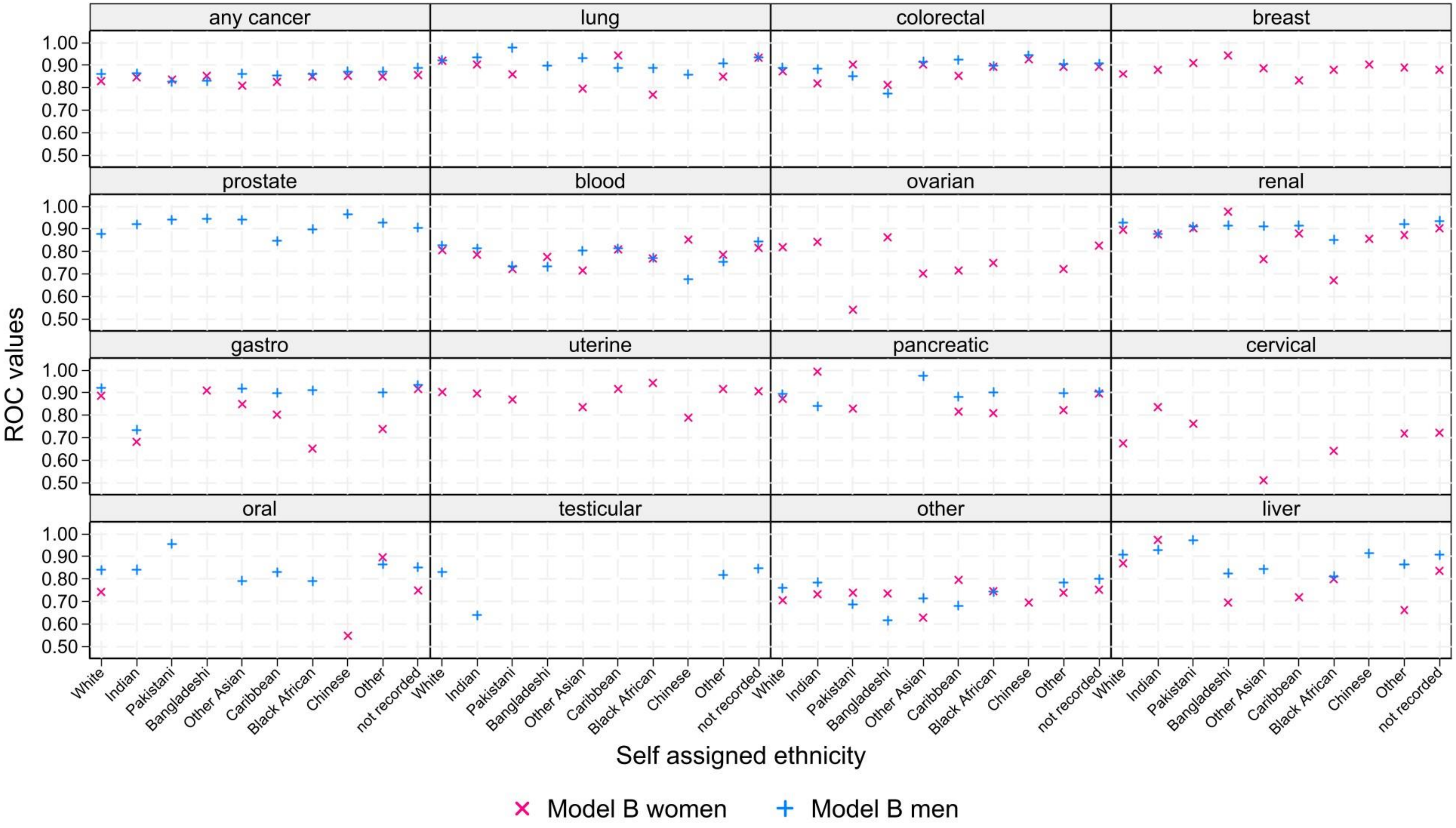

**Supplementary Figure 10. Discrimination values (c statistic) for final model A (without blood test) in men and women in the QResearch English validation cohort by age group (cells with less than 5 events suppressed).**

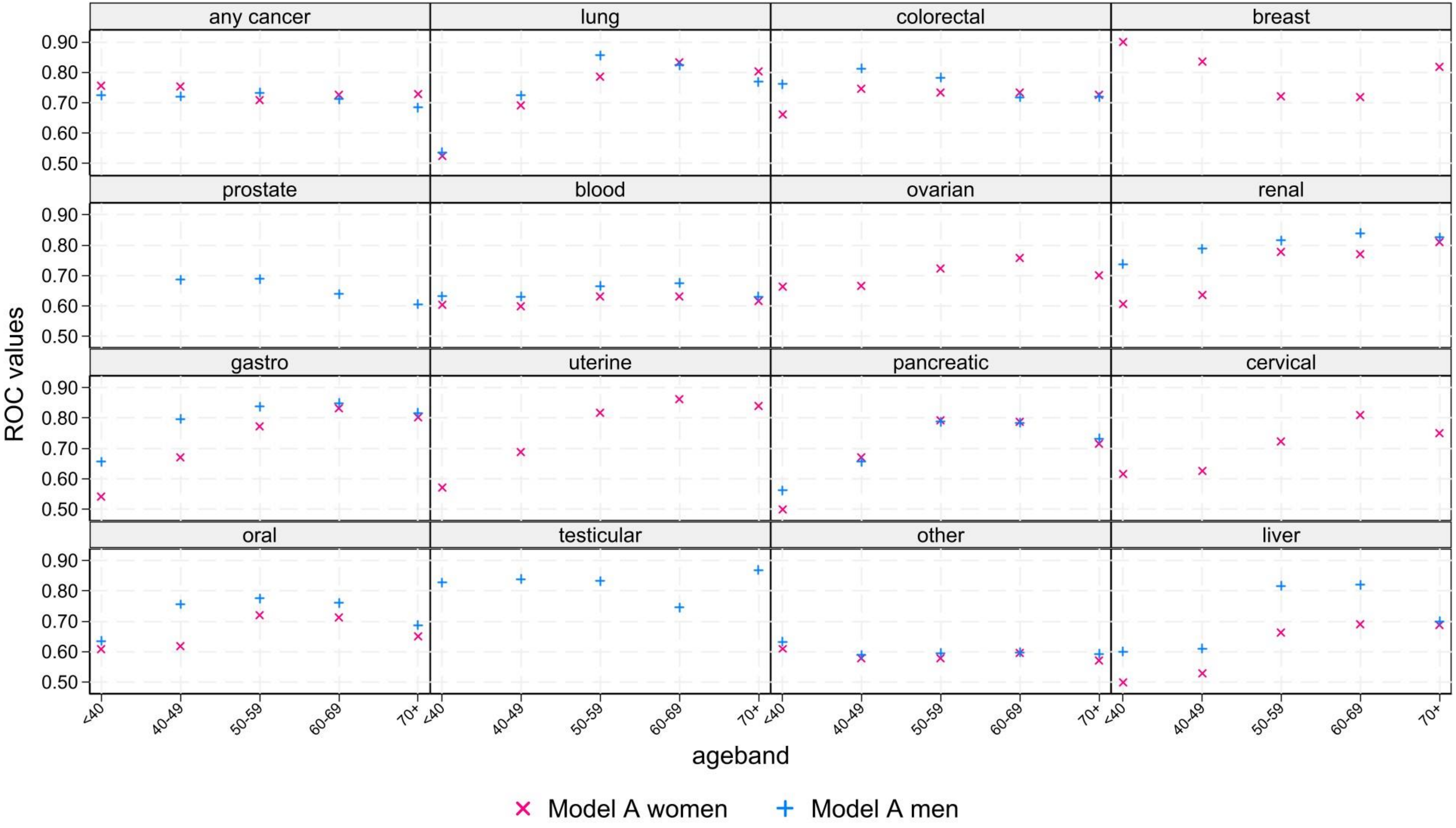

**Supplementary Figure 11. Discrimination for final model B (with blood test) in men and women in the QResearch English validation cohort by age (cells with less than 5 events suppressed).**

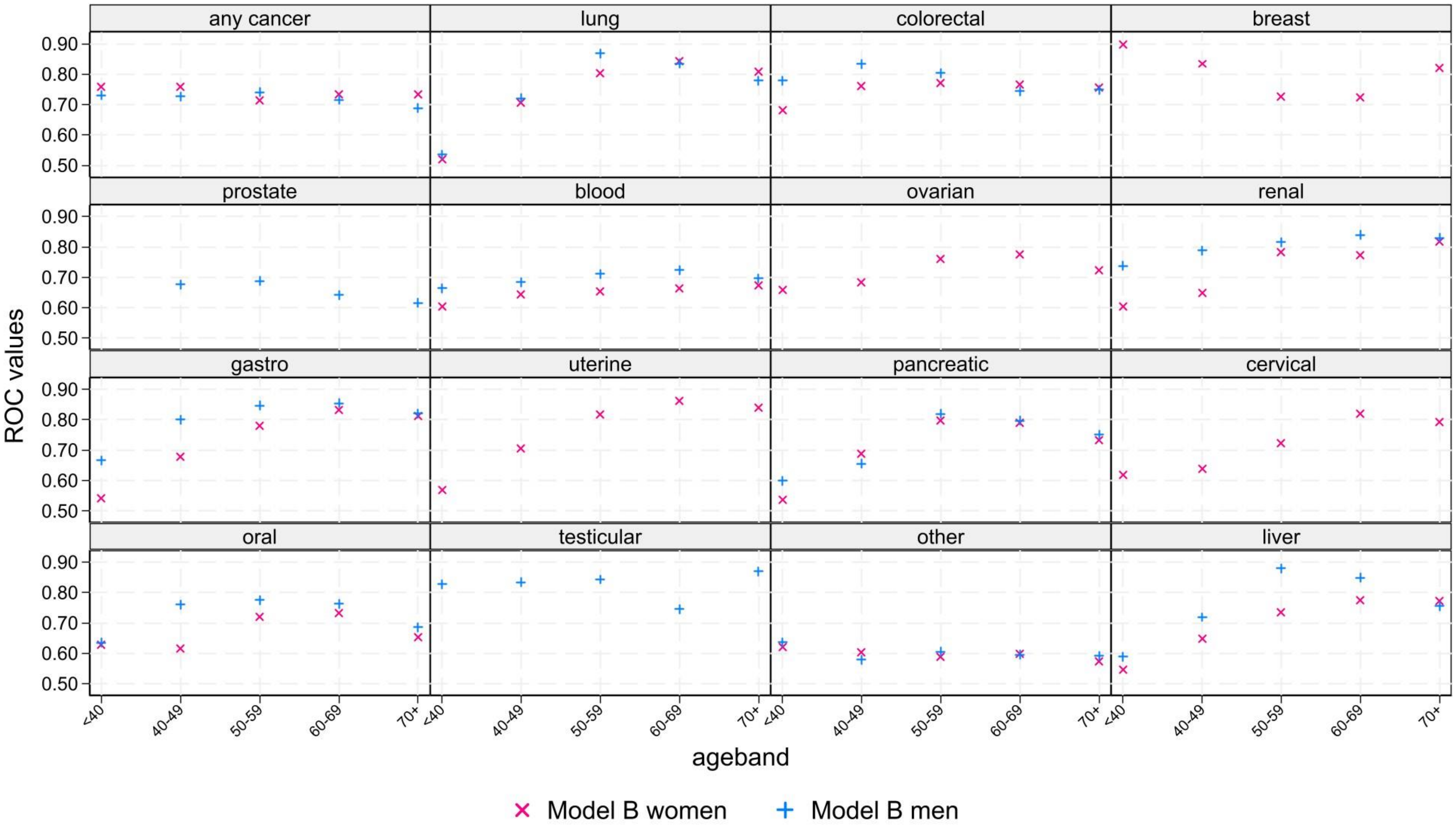

**Supplementary Figure 12. Discrimination for final model A (without blood test) in men and women in the QResearch English validation cohort by geographical region (cells with less than 5 events suppressed).**

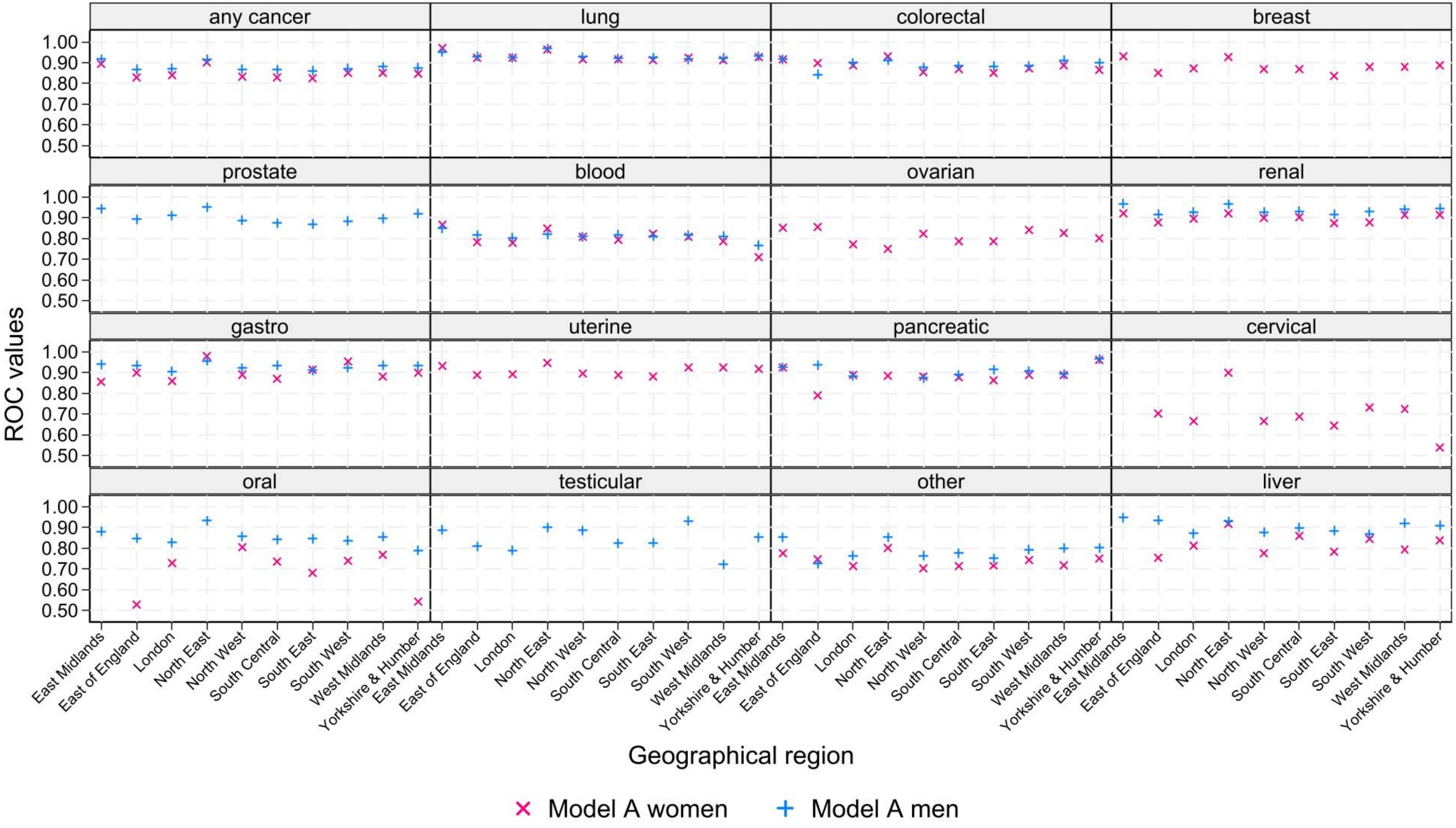

**Supplementary Figure 13. Discrimination for final model B (with blood test) in men and women in the QResearch English validation cohort by geographical region (cells with less than 5 events suppressed).**

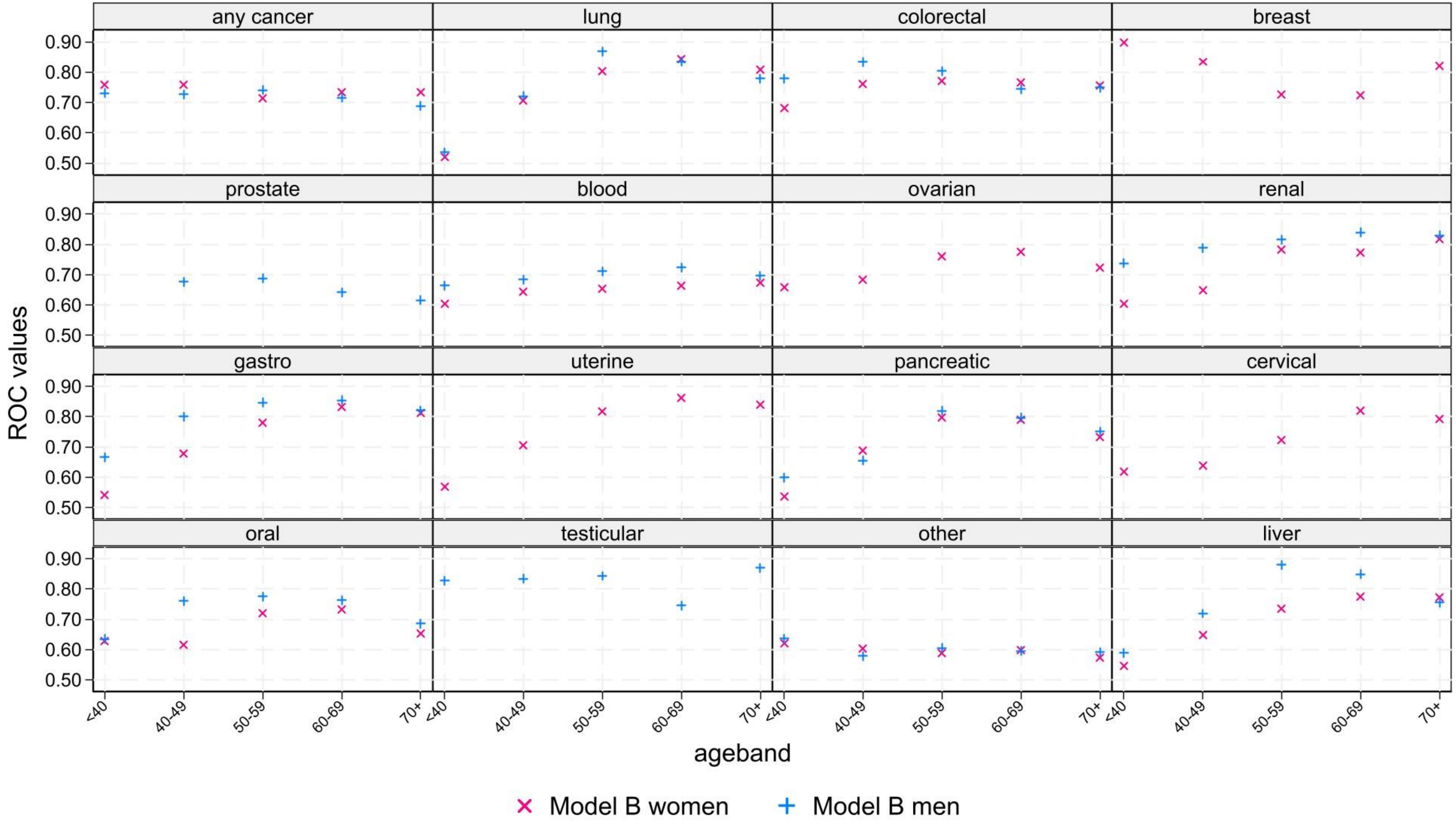

**Supplementary Figure 14. Predicted to observed probability for cancer overall in men and women comparing models A and B in people aged 18-84 in the QResearch English validation cohort and CPRD cohort from the devolved administrations.**

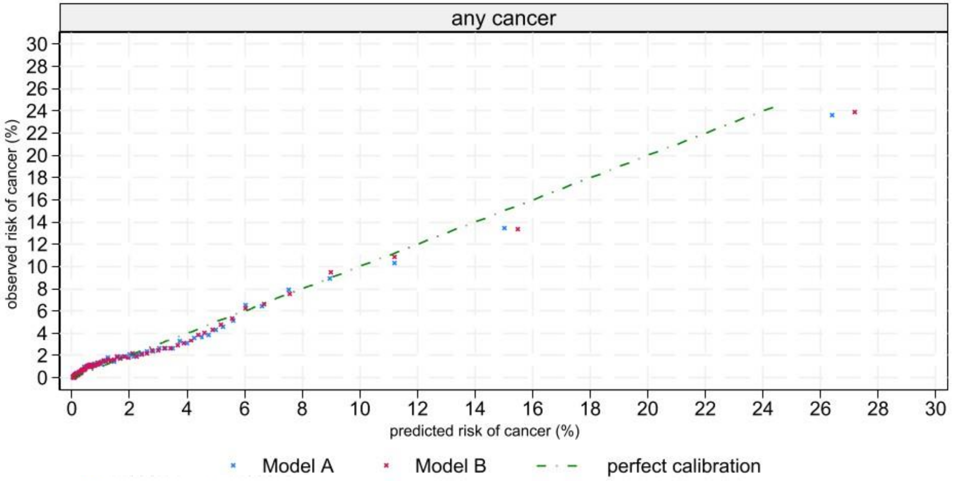


Calibration in women in England

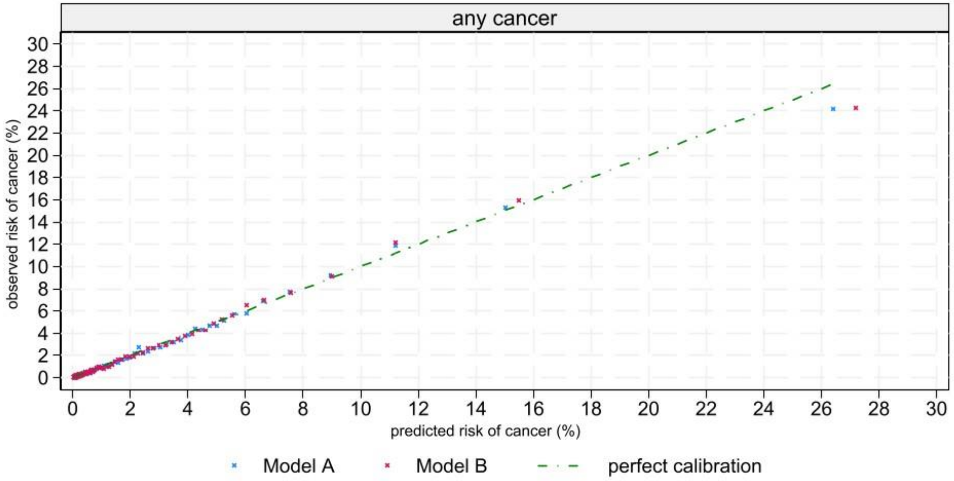


Calibration in men in England

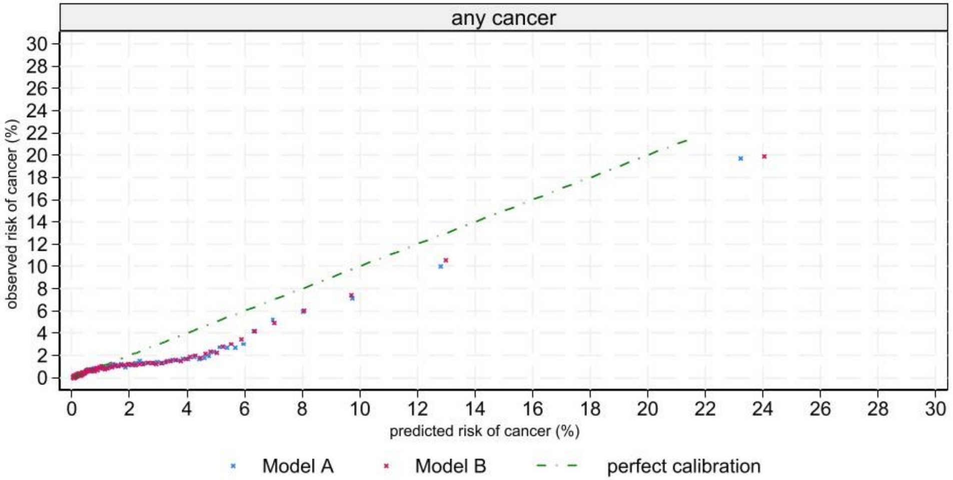


Calibration in women in devolved administrations

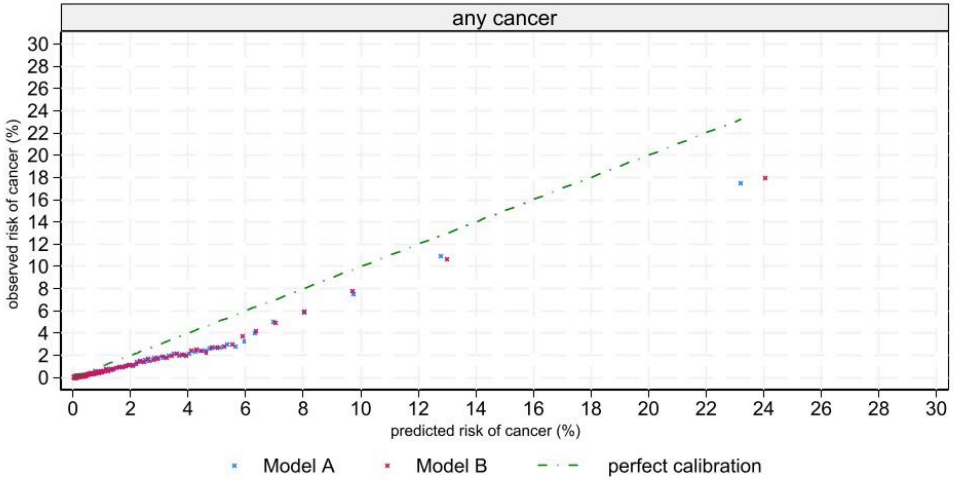


Calibration in men in devolved administrations

**Supplementary Figure 15. Predicted to observed probability for each cancer type in men and women comparing models A and B in people aged 18-84 in the English validation cohort.**

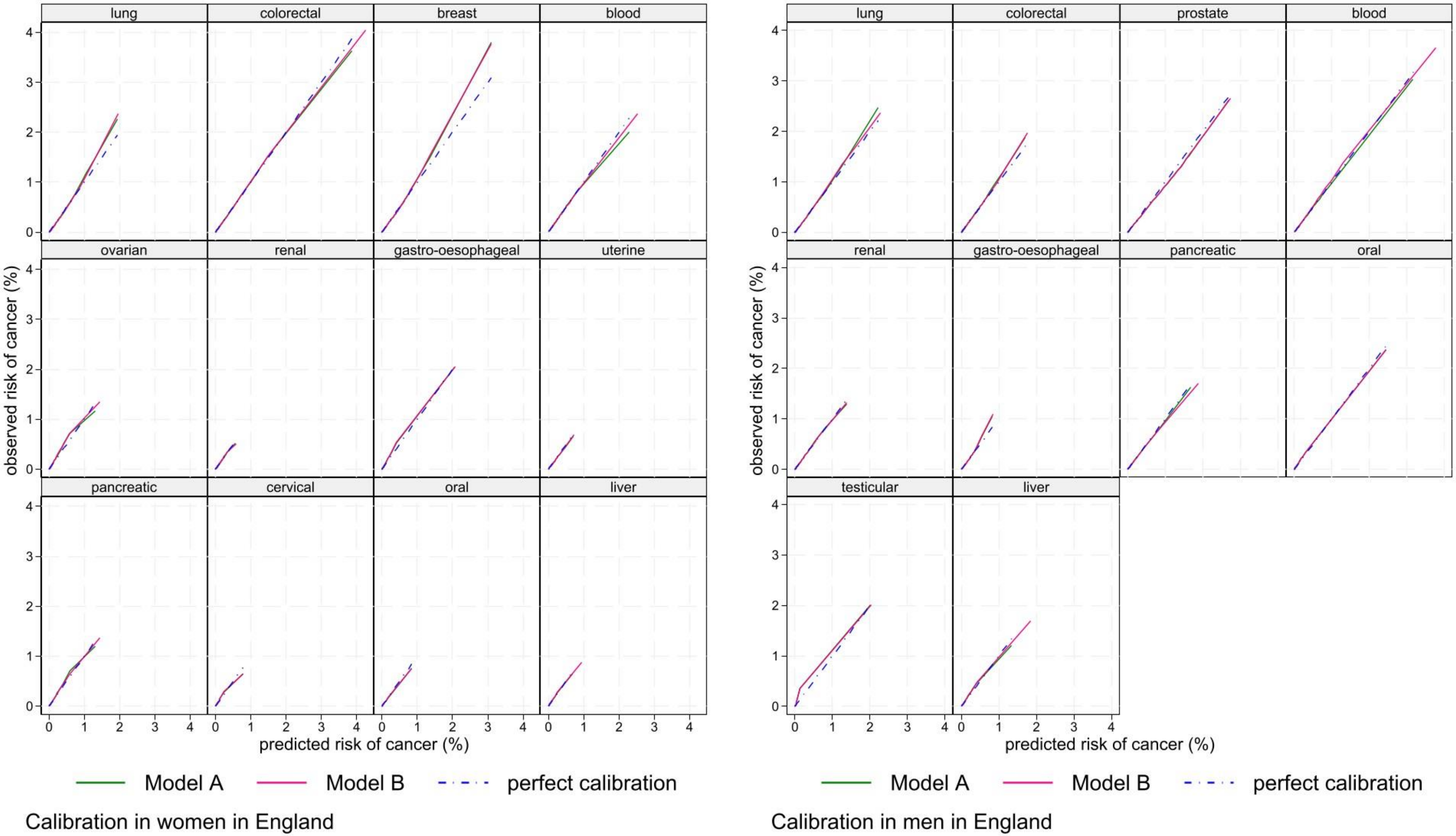

**Supplementary Figure 16 calibration plot showing predicted and observed probabilities for any cancer using QCancer in men and women aged 25-84 years in the QResearch English validation cohort and CPRD validation cohort in the devolved administrations.**

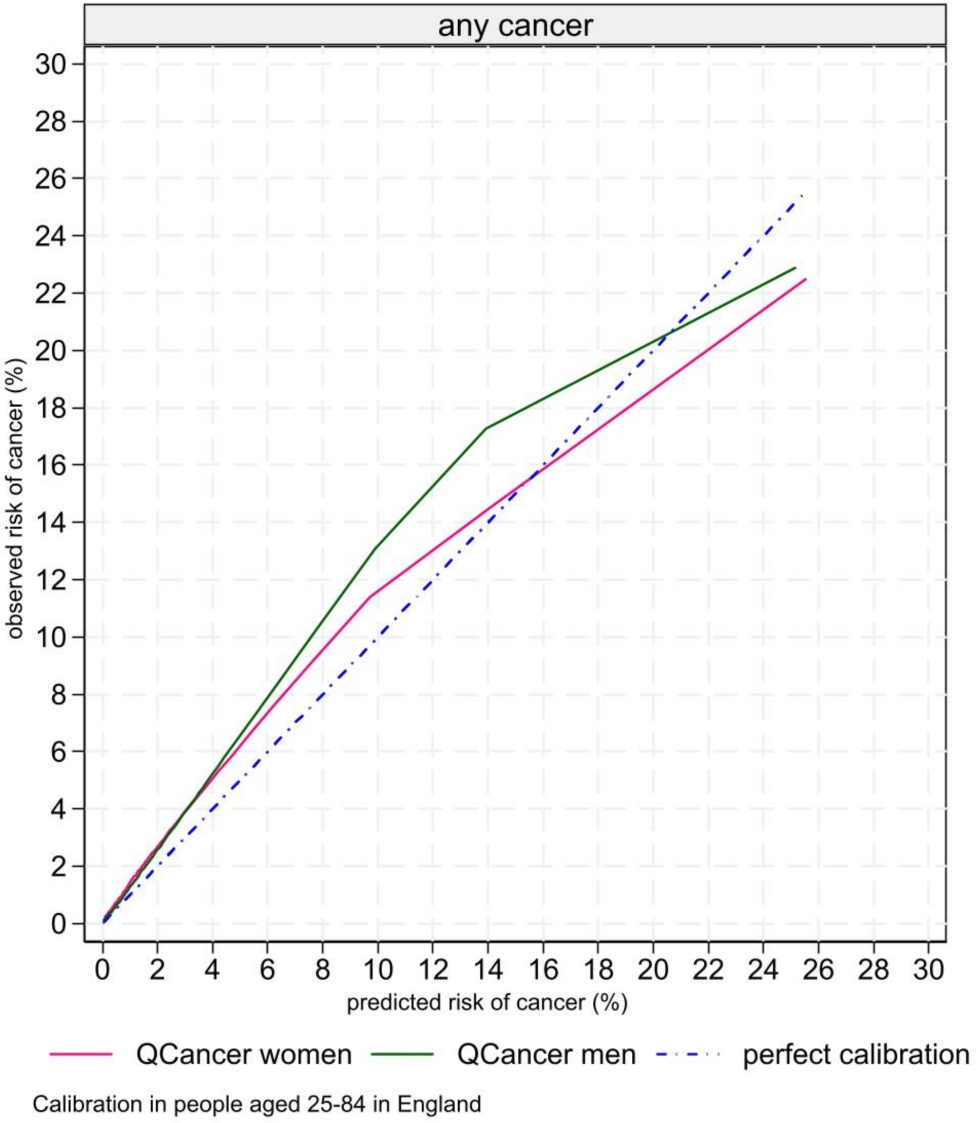


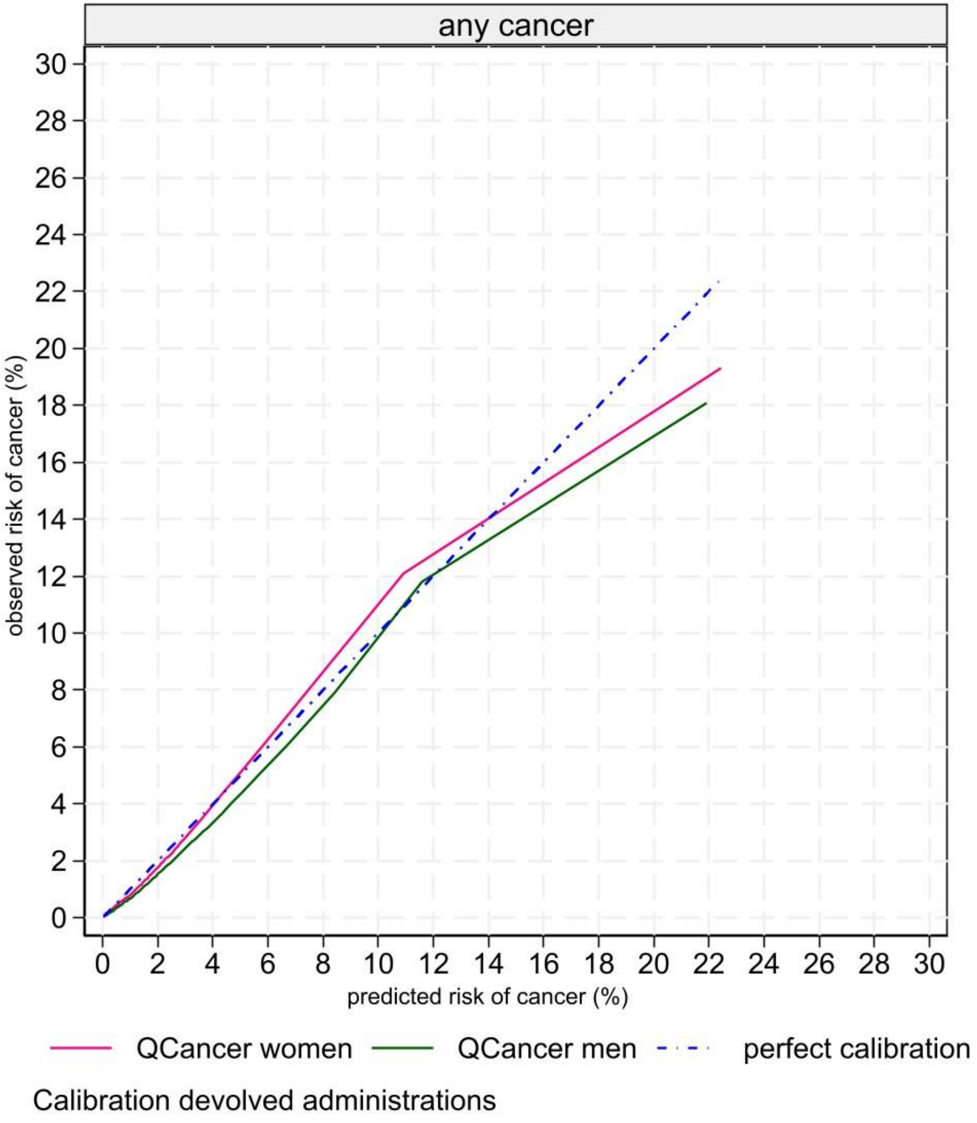

**Supplementary Figure 17 calibration plot showing predicted and observed probabilities by cancer type using QCancer in men and women aged 25-84 years in the QResearch English validation cohort.**

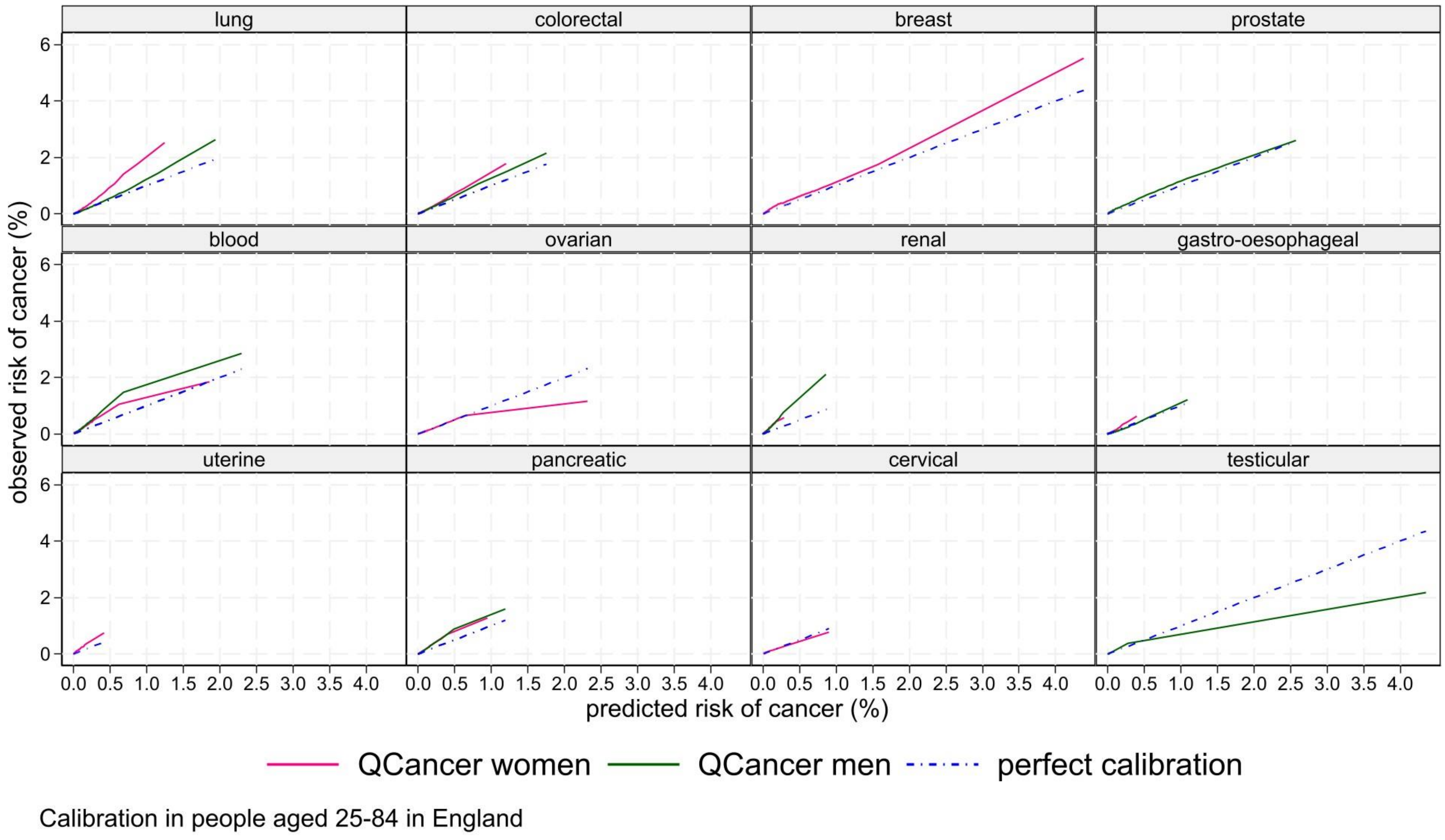

**Supplementary Figure 18 Decision analysis curves in patients aged 25-84 in the English validation cohort for any cancer vs no cancer using three models (QCancer, Model A, Model B).**

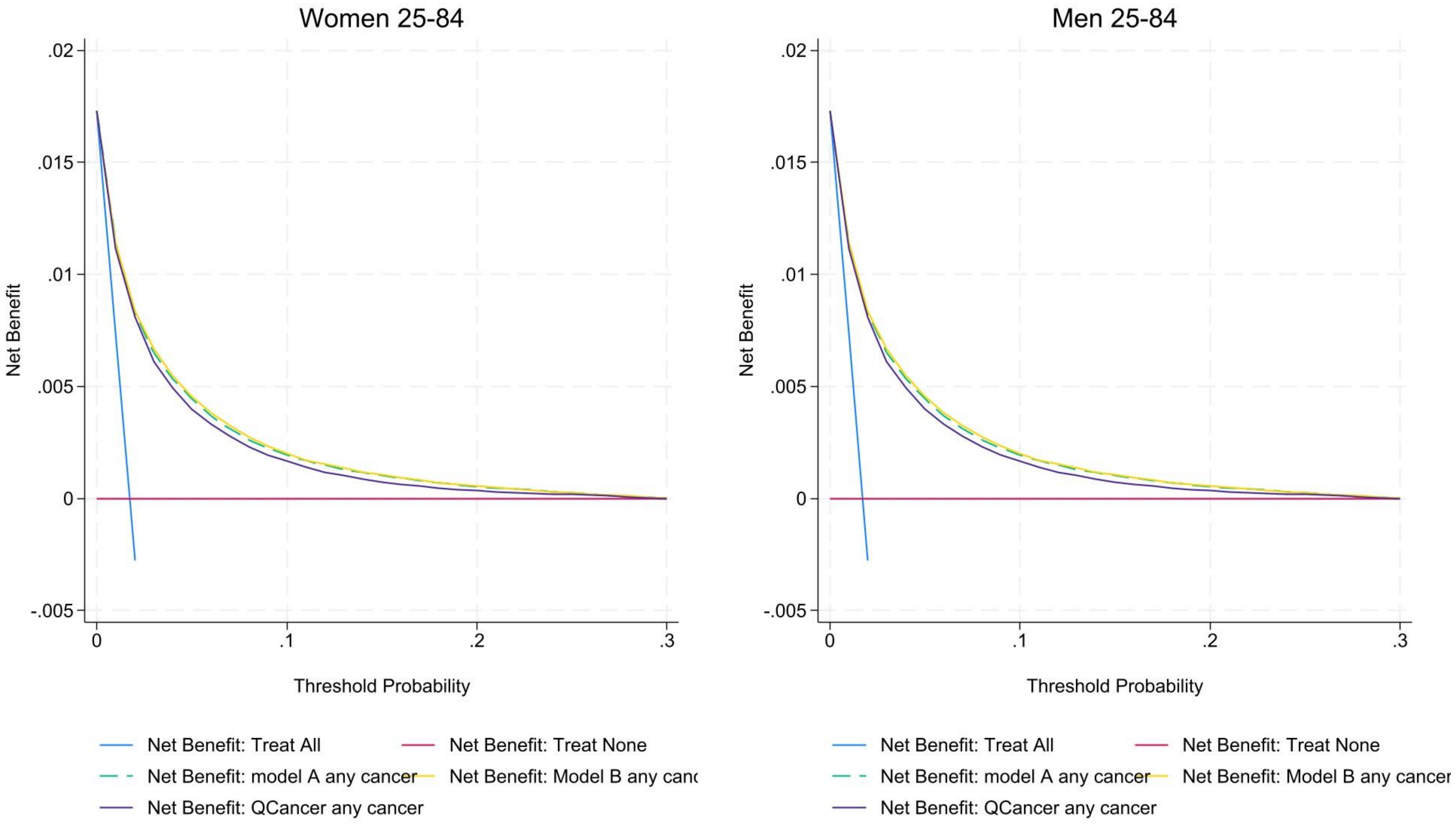

**Supplementary Figure 19 Decision analysis curves in patients aged 25-84 in the English validation cohort for lung cancer**

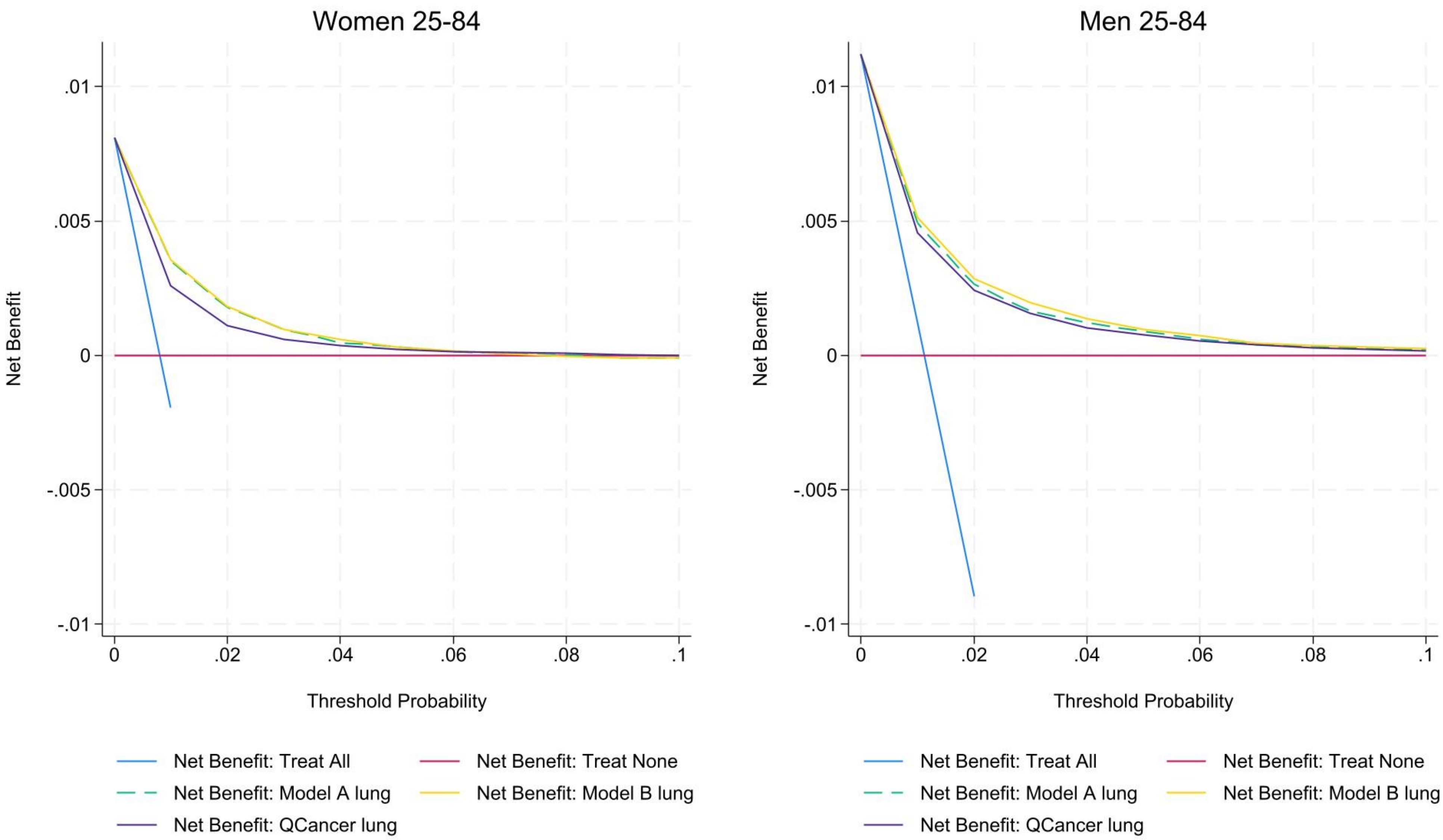

**Supplementary Figure 20 Decision analysis curves in patients aged 25-84 in the English validation cohort for colorectal cancer**

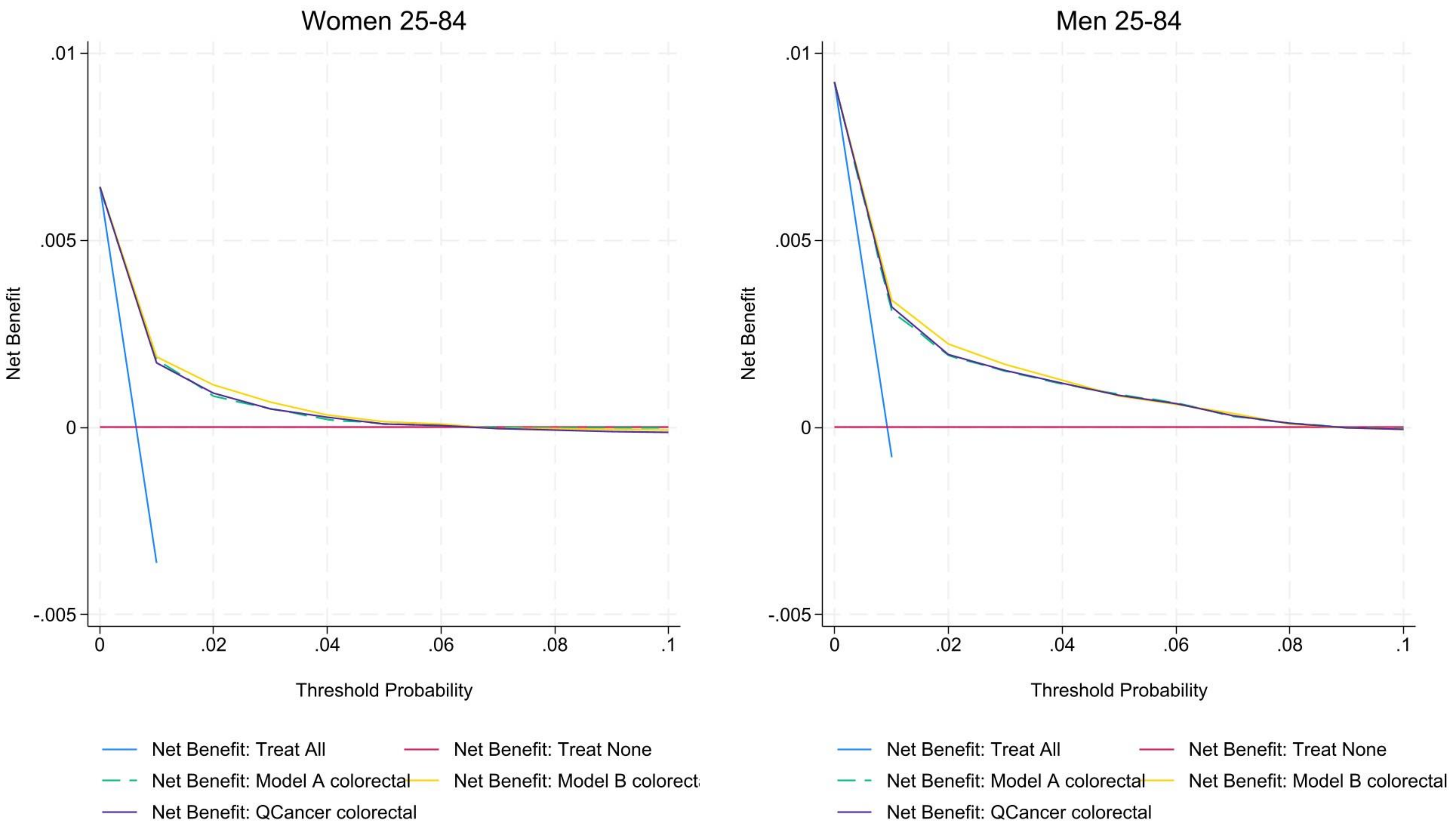

**Supplementary Figure 21 Decision analysis curves in patients aged 25-84 in the English validation cohort for blood cancer**

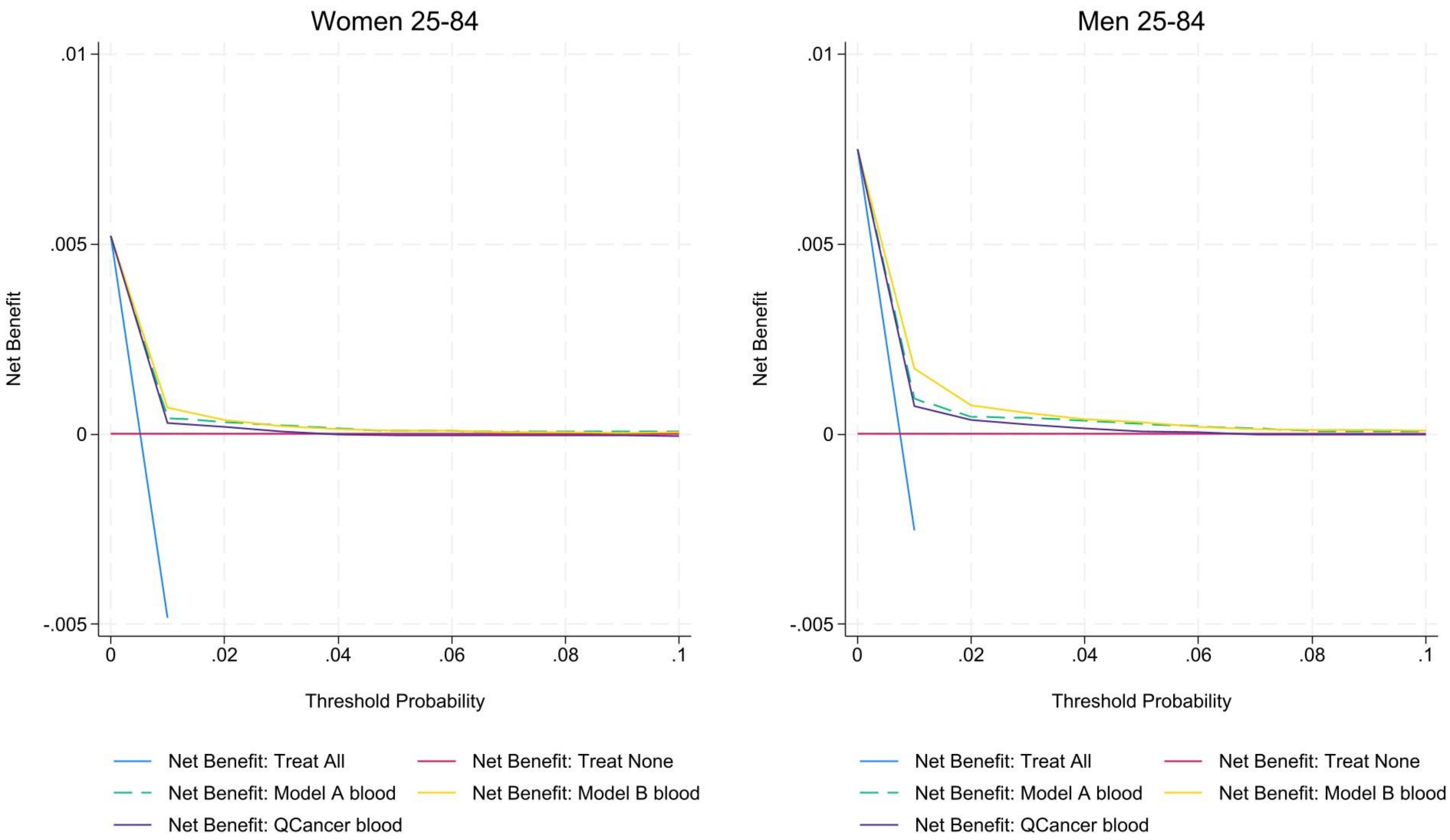

**Supplementary Figure 22 Decision analysis curves in patients aged 25-84 in the English validation cohort for renal cancer**

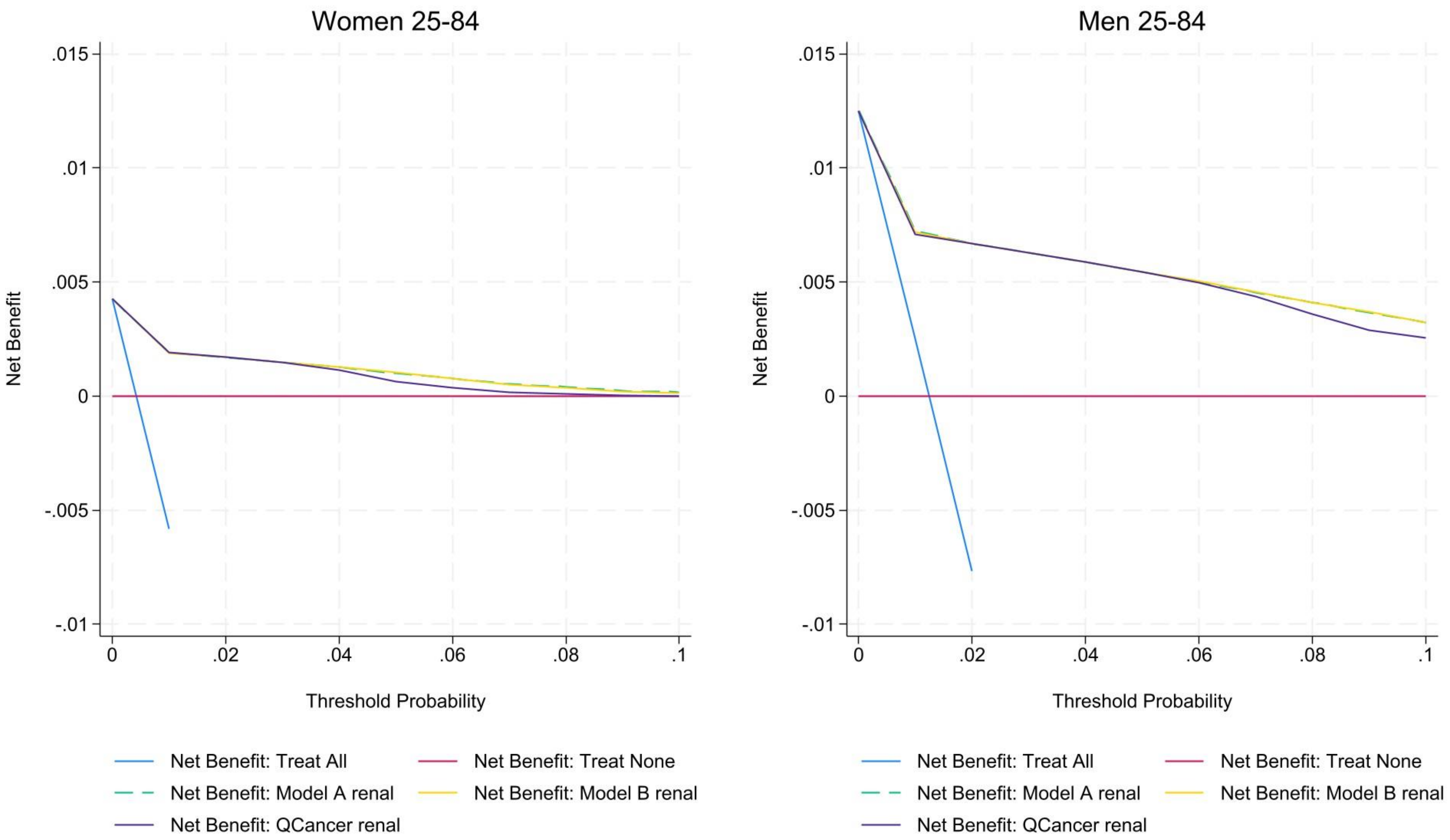

**Supplementary Figure 23 Decision analysis curves in patients aged 25-84 in the English validation cohort for gastro-oesophageal cancer**

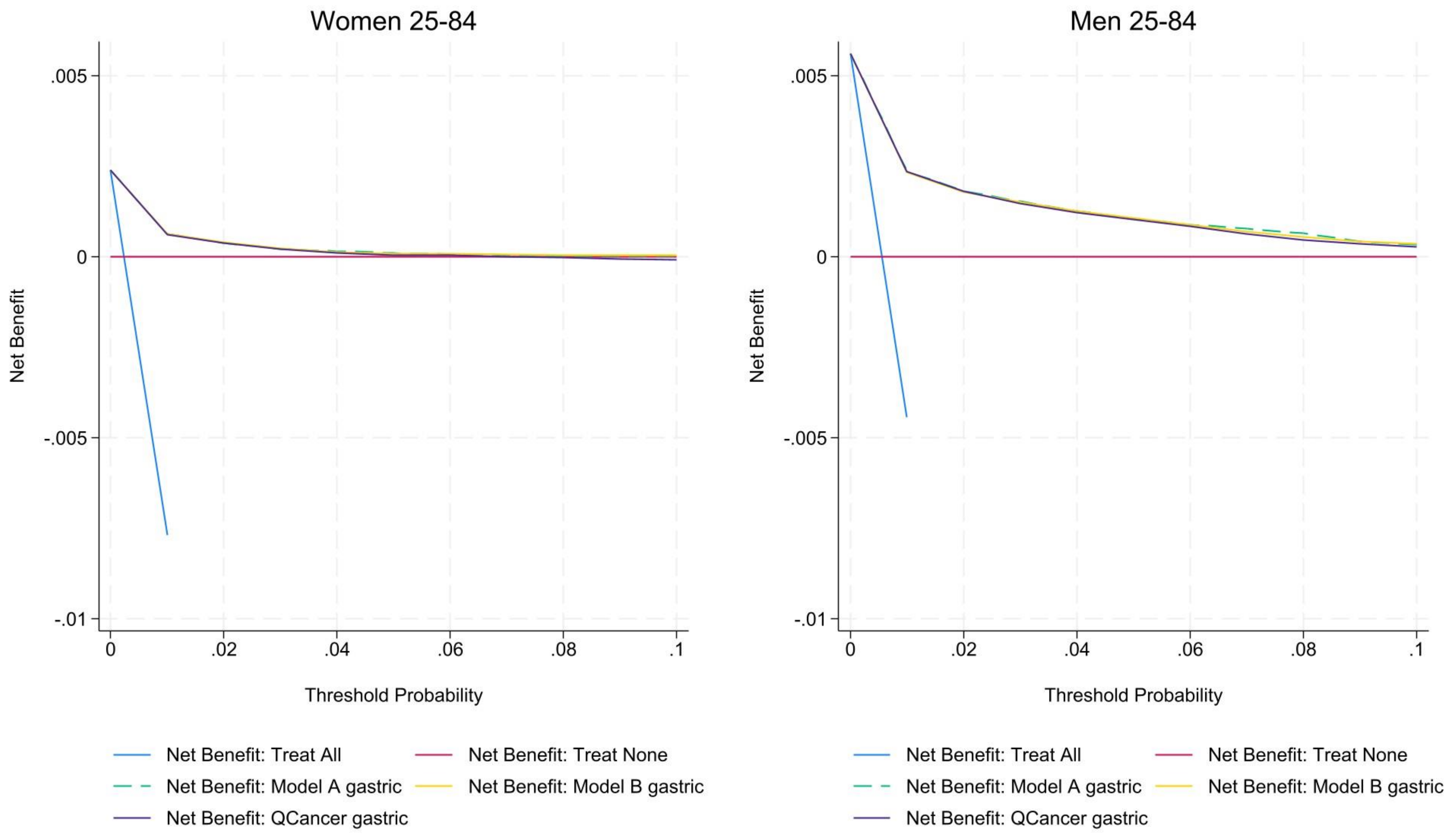

**Supplementary Figure 24 Decision analysis curves in patients aged 25-84 in the English validation cohort for pancreatic cancer**

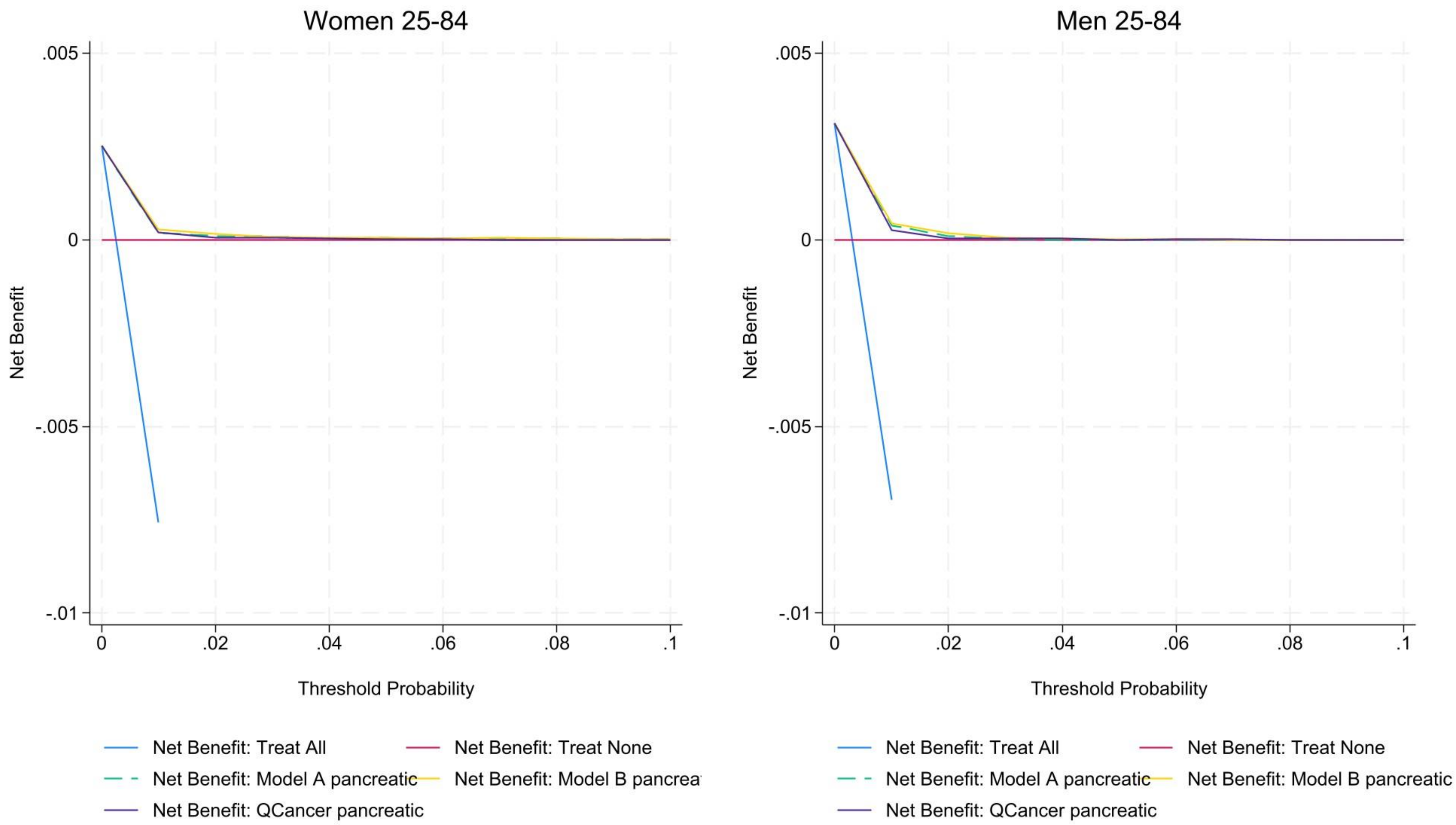

**Supplementary Figure 25 Decision analysis curves in women aged 25-84 in the English validation cohort for breast and ovarian cancer**

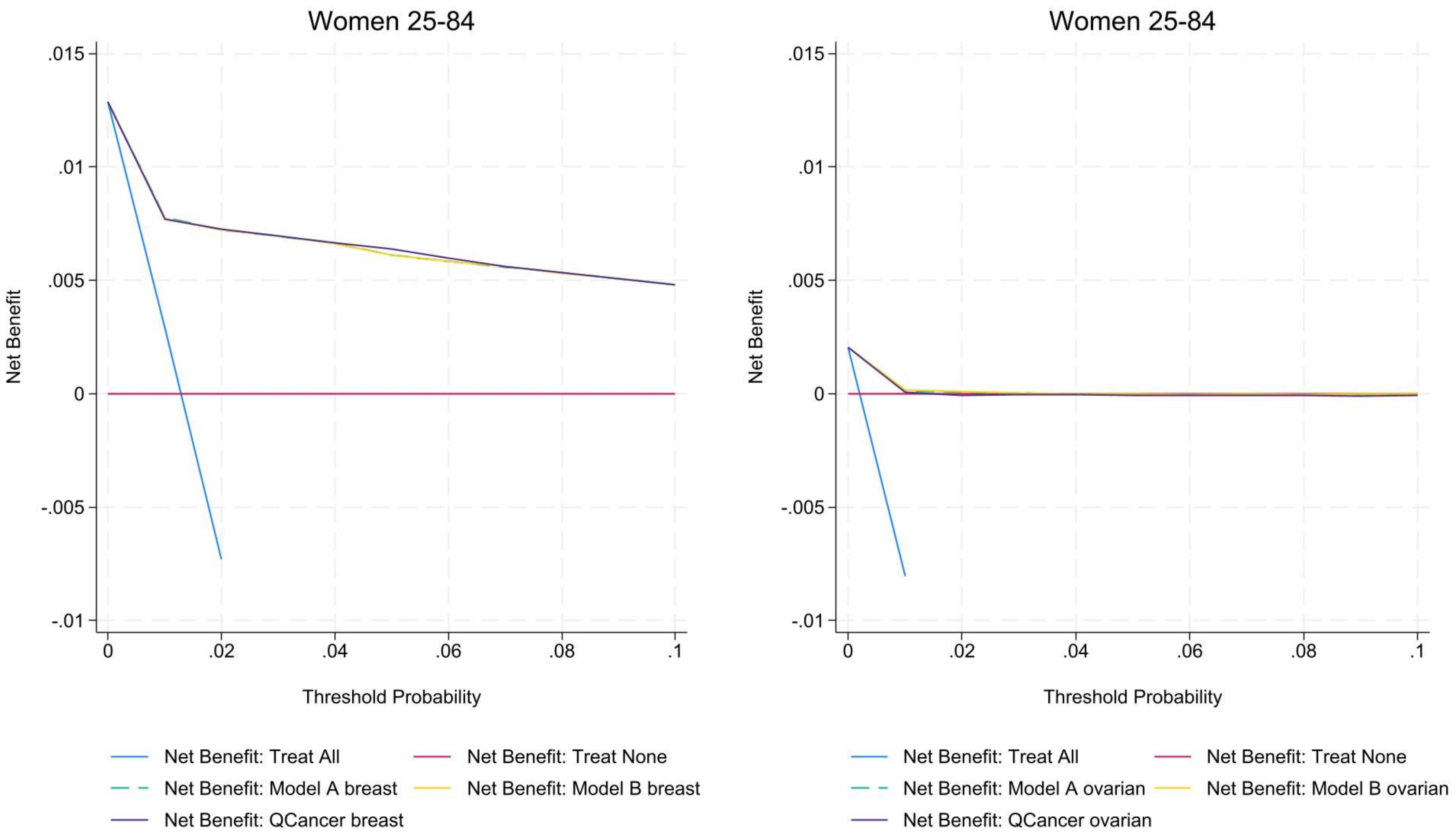

**Supplementary Figure 26 Decision analysis curves in aged 25-84 in the English validation cohort for uterine and prostate cancer.**

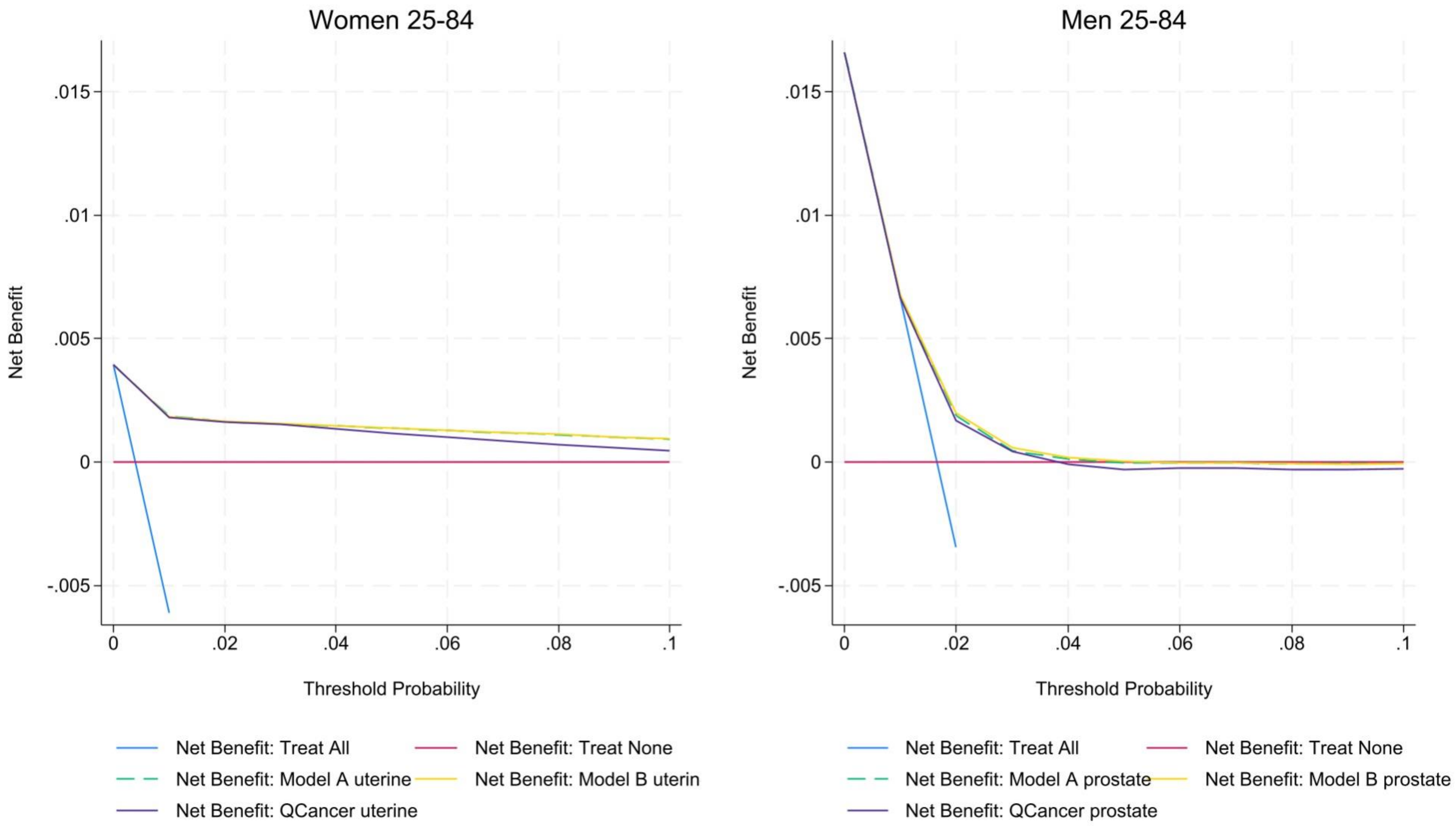

**Information on sample size calculations**

## 1. Model derivation sample size

The sample size of the derivation cohort was 3,622,789 for women, and 3,841,718 for men.

Using the sample size criteria for developing a multinomial prediction model in Pate et al[1], we determined the minimum sample size required based on a maximum of 100 predictor variables and the proportions with each type of cancer. For criterion 1, based on a target shrinkage value, we specified a shrinkage factor of 0.9. For criterion 2, targeting a difference in apparent and adjusted $R^2$ values, we used a maximum Cox-Snell apparent $R^2$ value of 0.22 (derived from equation 28) and a difference of 0.01, and for criterion 3 based on obtaining a precise estimate of overall probability, we used a margin of error value of 0.01.

For criterion 1 the minimum sample size required in women ranged from 88,764 to 1,159,042 for pairwise comparisons of individual cancer types with no cancer. For pairwise comparisons between cancer types the minimum sample sizes required for some of the more rare cancer pairs exceeded the sample size available, but following suggestions in the paper by Pate el al[1], by reducing shrinkage to 0.8 and the number of predictor variables to 60 for the two rarest cancers (oral and liver) the sample size required was 3,382,393. It should be noted that only a subset of the predictor variables were considered for inclusion in the model for some cancers with fewer relevant symptoms.

Similarly in men the minimum sample size required ranged from 134,076 to 926,911 for pairwise comparisons with no cancer. For pairwise comparisons between cancer types, for the two rarest cancers (testicular and liver), the sample size required was 3,227,602 for a shrinkage of 0.8 with 80 predictor variables.

For criterion 2 the minimum sample size required was 112,679 for women, and 115,859 for men.

For criterion 3 the minimum sample size required was 1479 for women, and 1422 for men.

Across these criteria the sample size of the available data met the required minimum sample sizes of 3,382,393 in women and 3,227,602 in men for the specified criteria, allowing for a reduced shrinkage target for the rare cancer pairs where there may be some overfitting.

## 2. Validation sample size

The sample size of the QResearch validation dataset was 1,277,015 for women, and 1,360,169 for men. The corresponding figures for CPRD were 1,373,006 for women and 1,363,720 for men.

We calculated the minimum sample size required for the validation analyses using the criteria in Riley et al[2] for validation of a prediction model with a binary outcome, as currently there are no specific criteria published for validation of multinomial prediction models. We calculated the minimum sample size required for comparisons of each cancer type against no cancer, and selected the highest of these.

We used the pmvalsampsize command in Stata[3], with target 95% confidence interval widths of 0.2 for the calibration in the large measure O/E (criterion 1), 0.2 for the calibration slope, and 0.1 for the C statistic based on a value of 0.8.

For criterion 1 the required sample size ranged from 68,271 for breast cancer to 1,281,174 for oral and liver cancer in women, and 116,121 for prostate cancer to 960,785 for testicular cancer in men.

For criterion 2 the required sample size ranged from 11,685 for breast cancer to 471,971 for oral cancer in women, and from 12,649 for prostate cancer to 303,678 for testicular cancer in men.

For criterion 3 the required sample size ranged from 13,492 for breast cancer to 250,492 for oral and liver cancer in women, and from 22,842 for prostate cancer to 187,888 for testicular cancer in men.

Across these criteria a sample size of 1,281,174 is required in women and 960,785 in men. The number in women slightly exceeded the number available in the cohort, based on criterion 1 for oral and liver cancers meaning the confidence interval for O/E will be expected to be slightly larger than 0.2 for these cancers.

**References**


1. Pate, A., *et al.* Minimum sample size for developing a multivariable prediction model using multinomial logistic regression. *Stat Methods Med Res*, 9622802231151220 (2023).
2. Riley, R.D., *et al.* Minimum sample size calculations for external validation of a clinical prediction model with a time-to-event outcome. *Statistics in medicine* (2021).
3. Joie, E. PMSAMPSIZE: Stata module to calculate the minimum sample size required for developing a multivariable prediction model. (Boston College Department of Economics, 2018).
